# On Inclusion of Covariates in Model Based Dose Finding Clinical Trial Designs

**DOI:** 10.1002/sim.10337

**Published:** 2025-01-24

**Authors:** Adrien Ollier, Pavel Mozgunov

**Affiliations:** ^1^ MRC Biostatistics Unit University of Cambridge Cambridge UK

**Keywords:** Bayesian analysis, Bayesian Lasso, BLRM, dose‐finding, variable inclusion

## Abstract

There is a growing number of Phase I dose‐finding studies that use a model‐based approach, such as the CRM or the EWOC method to estimate the dose‐toxicity relationship. It is common to assume that all patients will have similar toxicity risk given the dose regardless of patients' individual characteristics. In many trials, however, some patients' covariates (e.g., a concomitant drug assigned by a clinician) might have an impact on the dose‐toxicity relationship. In this work, motivated by a real trial, we evaluate an impact of taking into account (or omitting) some patients' covariates on the individual target dose recommendations and patients' safety in Phase I model‐based dose‐finding study. We investigate several variable penalisation criteria and found that, for continuous and binary covariates, omitting a prognostic covariate leads to a drastically low proportion of correct selections and an increase of overdosing. At the same time, including a covariate can lead to good operating characteristics in all scenarios but can sometimes slightly decrease the proportion of good selections and increase the overdosing. To tackle this, we propose to use a Bayesian Lasso Bayesian Logistic Regression Model (BLRM) and Spike‐and‐Slab BLRM. We have found that the BLRM coupled to the Bayesian LASSO and the BLRM with Spike‐and‐Slab are on average better appropriate to consider variable inclusion.

## 1 Introduction

One of the common aims of oncology dose‐finding clinical trials is to estimate the Maximum Tolerated Dose (MTD), that is the highest dose with a probability of Dose‐Limiting Toxicity (DLT) lower than a pre‐defined threshold. The dose‐finding methods in those trials are usually sequential, the results in the previous patients determining the dose for each patient in the new cohort according to a pre‐specified decision rule. Several methods have been proposed to perform those trials, for example parametric approaches such as logistic models [1] or the Continual Reassessment Method (CRM [2]) in which the dose‐toxicity relationship is modelled through a logistic regression. Extensions of those methods permit to take into account additional information during the clinical trial. For instance, the Time‐to‐event (TITE) CRM [3] was proposed to accommodate with delayed toxicities, while some models recently proposed permit to include some pharmacokinetics data [4].

Increasingly clinical trials consider a personalised treatment approach for the patients [5]. Specifically, in dose‐finding trials, the perspective of variable selection has been explored with a attention given to biomarkers for toxicity and/or efficacy [6, 7, 8]. Variable inclusion for overdose control with one covariate (continuous or binary) was investigated by Chen et al. [8]. Otherwise, inclusion of one continuous covariate in an EWOC model was considered by Babb and Rogatko [9] in a particular dose‐finding trial to adjust each patient dose level taking into account pre‐treatment concentration of an antibody used to moderate the effect of the agent being escalated, which permited to define each patient's individual MTD.

The possibility of taking into account ordered subgroups of patients was investigated [10, 11]. In particular, O'Quigley and Paoletti [10] included a shift parameter between groups in their model. Another approach proposed by Cotterill and Jaki [12] compared several priors for a regression approach including two pre‐specified subgroups. In particular, an additional term is considered to model one subgroup dose‐toxicity relationship, and several prior distributions were investigated, included prior distributions defined using pseudo‐observations as described by Whitehead and Brunier [13], or Spike and Slab prior distributions. Otherwise, subgroup approaches were also investigated for phase I‐II clinical trials. A subgroup approach was investigated by Thall, Nguyen, and Estey [14] using historical data to obtain information on covariates but without variable penalisation consideration. Time‐to‐event toxicity considering subgroups was investigated by Chapple and Thall [15].

In this context, the concept of personalised Maximum Tolerated Dose (pMTD) or individual dosing was introduced by several authors [8, 16]. Most of the methods proposed to evaluate their performance either in term of parameter estimation [8, 16] or selection rate for the target dose [7] To select covariates, the Least Absolute Shrinkage and Selection Operator (LASSO [17]) method is often used, as well as its extension the Bayesian LASSO [18, 19]. This method was used by Kakurai et al. [7] in the context of phase I‐II dose‐finding trial with bivariates binary outcomes (toxicity and efficacy) using binary covariates, taking into account both the toxicity and the efficacy in the dose‐finding trial. The authors notably used a criterion based on the posterior distribution of the covariates' coefficient to perform variable selection at the end of the trial.

The methodology proposed in this manuscript concerns the dose‐finding trials *in patients*, i.e., the setting where the patients are recruited in small cohorts of patients (3–5) to several doses of an experimental drug after the outcomes for the previous cohort is evaluated. The focus of this studies is primarily on the safety and tolerability of the experimental agent. This setting originates in oncology trials, in which, the experimental drug will be given to the cancer patients, but is also increasingly applied in non‐oncology settings. For example, related model‐based dose‐finding designs have been recently applied in infectious diseases, specifically, tuberculosis [20] and COVID‐19 [21], in anaesthesiology [22, 23], neurological diseases [24], and opioid detoxification [25] (our motivating trial). As the result, the methodology covered in this manuscript can be applied in the related dose‐finding settings in patients. However, to streamline the presentation we will employ the terminology commonly used in oncology setting, for instance we will be referring to DLT or MTD.

The methods described above were either proposed into a very specific clinical context [9] or considering subgroups through binary covariates or ordered subgroups. When models considering penalisation were proposed, it was in the setting of subgroups [7, 12]. The issue of the model performance when not including a significant variable was studied with the EWOC model [9], as well as the MTD estimation in the EWOC model considering covariate. However, the questions of including a non‐significant variable while comparing several regression models with or without penalisation for continous covariates coefficient is not treated, neither the issue of patients allocation to their pMTD during the trial and the good pMTD recommendation at the end of the trial.

In this work we propose and evaluate several approaches to handle covariates and individual MTD recommendation in dose‐finding trials. We evaluate the impact of taking (or not taking) into account a significant variable for the pMTD's proportion of good selection, but also the impact of including a non‐significant variable during the dose‐finding trial. To do so, we investigated several methods to consider the covariates during the trial, some models proposed here consider a penalisation for the covariate coefficient, whatever the covariate is binary or continuous. We also evaluate the consequences of taking into account non‐significant covariates in the models during the trial, considering two normal covariates, one binary and one continuous. We notably used the Bayesian Logistic Regression model (BLRM [26]) and extend it to include covariates. Several prior distributions for the covariates' parameter were evaluated, including the Bayesian LASSO and Spike and Slab priors [27].

This paper is organised as follows. In Section 2 is presented a motivating case study, in Section 3 the method is presented in detail, a simulation study is described in Section 4, with its results in Sections 5 and 6. The article concludes with a discussion.

## 2 Motivating Case Study

This work is motivated by the clinical trial originally design by Mozgunov et al. [25] which is a trial of an opiate detoxification trial [28] in patients receiving methadone, an opioide substitute. To overcome withdrawal symptoms, baclofen was investigated for patients receiving methadone. The trial consisted of two parts: Study 1 was a dose‐finding trial to assess the safety of baclofen in combination with methadone, followed by Study 2 which was by a randomised controlled proof‐of‐concept study. The particularity of this trial is the patients received a continuous dose of methadone prior to the trial.

The aim of Study 1 was to estimate a *dosing function* to recommend a dose of baclofen (among a range of four) with a probability of dose‐limiting toxicity (DLT) in 15%–25% given the individual dose of methadone that the patient receives. Therefore, it was a combination dose‐finding trial with the doses of baclofen only being escalated. The clinical team assumed that the dose of methadone might influence the probability of DLT in the combination with baclofen. This trial was, therefore, a case where a dose‐finding should account for one continuous covariate: the externally defined dose of methadone. The trial used a model‐based Bayesian design to guide the dose‐finding decisions with the model directly including the methadone covariate. The coefficient for the methadone covariate was treated similarly to the coefficient to the baclofen‐toxicity relationship, and no penalisation or variable selection procedure was considered.

While the clinical team was more certain about the impact (and hence inclusion) of the methadone dose covariate, there were also other factors discussed that could have an impact on the toxicity risk. These included, for example, gender, age, and weight. However, given a small study sample size, study timeline, and the unknown effect of including many (possibly non‐significant) covariates in a dose‐finding trial, the decision was made to include only one covariate in the model and use others for the Dose Setting Committee outside of the model. In this work, we tackle the question of whether a model‐based design could have provided appropriate guidance for the dose‐finding decision considering covariates.

## 3 Method

### 3.1 Setting

We consider a phase I clinical trial single‐agent treatment with a binary outcome Y for toxicity, $y_{i}=1$ if patient i experienced a Dose‐Limiting‐Toxicity (DLT) and $y_{i}=0$ if not. In contrast to a conventional dose‐finding study that aims to identify the single MTD for all patients, the objective of the considered setting is to identify the pMTD given patients' personal characteristics (covariates); in the cases where one or several covariates should be taken into account, not a single MTD will be identified at the end of the trial but the pMTD for any patient given their covariates at the end of the trial.

### 3.2 Model‐Based Dose‐Finding Designs

The designs presented hereafter are model‐based designs. In parametric models for dose‐finding designs, the dose‐toxicity relationship through a parametric model. This allows to use of all cohorts' information in the estimation of the dose‐toxicity relationship.

#### 3.2.1 BLRM Design

In conventional dose‐finding models, only the dose is considered, and no covariates are taken into account. Where no covariates are considered during the trial, the BLRM model [13] can be used to model the dose‐toxicity relationship:

(1) $$\text{logit}\left(p_{T}(d_{k})\right)=\alpha +\beta \log(d_{k}/d_{r})$$

where $p_{T}(d_{k})$ is the probability of DLT for a given dose $d_{k}$ and $d_{r}$ are reference doses. The vector of parameters of interest is θ=(α,log(β)), log(α) being the intercept and β the slope for the log‐dose, and the prior distribution π(θ) for θ is a bivariate normal distribution:

(2) $$\left(\begin{array}{l} \alpha \\ \log(\beta ) \end{array}\right)∼ℳ𝒩\left(\left[\begin{array}{l} m_{1}\\ m_{2} \end{array}\right],\left[\begin{array}{ll} \sigma_{11}^{2} & \sigma_{12}\\ \sigma_{21} & \sigma_{22}^{2} \end{array}\right]\right)$$

This bivariate normal prior distribution for intercept and slope is a widely used one and does not consider any penalization for the coefficients or variable selection procedure.

The likelihood function after n patients can be written

(3) $$L(\theta |D_{n})=\overset{n}{\underset{i=1}{\prod }}(p_{T}{\left(d_{\left(i\right)}\right)}^{y_{i}}{\left(1-p_{T}(d_{\left(i\right)})\right)}^{1-y_{i}}$$

where $d_{\left(i\right)}$ is the dose given to the ith patient, $y_{i}$ the ith patient's binary outcome, and $D_{n}$ the outcomes of the first n patients. The posterior estimates are obtained using the Bayes theorem

(4) $$\pi (\theta |D_{n})=\frac{L(\theta |D_{n})\pi (\theta )}{\int_{{\mathbb{R}}^{2}}L(\theta |D_{n})\pi (\theta )d\theta }$$

To perform the dose‐finding process, we chose to adopt a similar approach to model‐based designs, in which patients are sequentially allocated to the highest dose $d_{i^{*}}$ associated to the closest target toxicity τ:

(5) $$i^{*}=\underset{k\in \{1,\ldots ,K\}}{\text{argmin}}|\hat{p_{T}}(d_{k})-\tau |$$

where $\hat{p_{T}}(d_{k})$ is an approximation of $𝔼[p_{T}(d_{k})]$ calculated using the posterior distribution of θ. The dose allocation does not take into account any covariates here.

### 3.3 BLRM With Covariates (Blrmc)

We here consider a straightforward extension of the BLRM that permits including additional covariates. We denote $X=[X_{ij}]$ a n×p matrix of patients' covariates and $X_{i•}$ the ith line of the matrix, that is the ith patient's vector of p covariates $X_{i•}=(x_{i1},\ldots ,x_{ip})$. For the ith patient, the probability of toxicity at dose $d_{k}$ is

(6) $$\text{logit}\left(p_{T}(d_{k}|X_{i•})\right)=\alpha +\beta \log(d_{k}/d_{r})+\overset{p}{\underset{j=1}{\sum }}\gamma_{j}x_{ij}$$

where $p_{T}(d_{k}|X_{i•})=\mathbb{P}(Y_{i}=1|d_{k},X_{i•})$ is the probability of DLT for patient i at dose $d_{k}$ and $\gamma_{j}$ the coefficient for the jth covariate. The vector of parameters of interest is $\theta =(\alpha ,\log(\beta ),\gamma_{1},\ldots ,\gamma_{p})$. The same normal prior distributions as in the BLRM are used for the intercept and slope, as well as for the $\gamma_{j}$ parameters: $\gamma_{j}∼𝒩(0,\sigma^{2})$.

The posterior distribution is obtained similarly to the BLRM (Equation (4), where $D_{n}$ denotes the outcomes and covariates of the first n patients), with the only difference being that the distribution of gamma is also updated. When one covariate is included, the method is named BLRMc1, while it is named BLRMc2 when two covariates are included.

For the dose‐allocation rule, we chose to adopt a similar approach to the one used for the BLRM. For each following cohort, the patients were individually allocated to their estimated pMTD $d_{i^{*}}$:

(7) $$i^{*}=\underset{k\in \{1,\ldots ,K\}}{\text{argmin}}|\hat{p_{T}}(d_{k}|X_{i•})-\tau |$$

where $X_{i•}$ denotes the vector of covariates for patients i, $\hat{p_{T}}(d_{k}|X_{i•})=\mathbb{P}(Y_{i}=1|d_{k},X_{i•})$ is the estimated probability of DLT for patient i at dose $d_{k}$ calculated with one of the regression models described in Section 3.2, using the data and outcomes of patients already included in the trial.

### 3.4 Blrmc With Variable Inclusion Criterion

It is possible, instead of directly including the covariate since the beginning of the trial, to consider a variable inclusion criterion. To perform variable inclusion, we proposed the $c_{\text{change}}$ criterion. The proposed criterion includes the covariate if its inclusion implies a significant change in dose allocation for the patients during the trial. More precisely, the trial starts using the BLRM method without covariates; however, the individual covariates are stored at each cohort. After a cohort of patients, the sequence of pMTDs for the previous and the immediate next cohort is calculated: we denote $d_{\left(i,n\right)}^{BLRM}$ the dose the patient i should be allocated to after n patients according to the BLRM model, and $d_{\left(i,n\right)}^{BLRMc}$ the dose the patient i should be allocated to after n patients according to the BLRMc model. If the proportion ℛ of differences between the two sequences is higher than a threshold, the covariate is included:

(8) $$ℛ=\frac{1}{n}\overset{n}{\underset{i=1}{\sum }}I\left(d_{\left(i,n\right)}^{BLRM}\neq d_{\left(i,n\right)}^{BLRMc}\right)>c_{\text{change}}$$

with I being the indicator function and $c_{\text{change}}$ the threshold to consider inclusion. If we want to consider variable inclusion during the trial, the trial starts with the BLRM, and at the end of each cohort, the $c_{\text{change}}$ criterion is applied. If the covariate is not included, the next cohort is allocated the estimated MTD using the BLRM model, whereas if included, each patient of the next cohort of patients will be allocated to its estimated pMTD using the BLRMc model. Once the covariate is included, if the criterion is reached, it remains until the end of the trial. This is to avoid changing the model several times during the trial, which could lead to confusion in interpret whether the covariate should be included or not. In the case of more than one covariate, the proportion ℛ is evaluated separately for each covariate, and then, each covariate can be included separately if higher the threshold $c_{change}$. It is then possible to include just one covariate if the others do not reach the threshold, or no covariate, or all if all covariates have a ℛ higher than the threshold.

For the following models with covariates, the intercept and slope for dose always kept the same prior distributions as in the BLRM (2), and only the covariates' part was modified.

The extension of the BLRM to the covariates handles the covariate coefficients similarly to the intercept and slope (i.e., the dose coefficient) this is reflected via a similar parametric form of the prior distribution on the corresponding coefficients. While the dose is known to have an effect on the toxicities, there could be more uncertainty on the covariates. To reflect this and to allow the coefficients for the covariates to be shrunk to 0, different types of the prior distribution for the covariate coefficients could be used. The design proposal presents two of such approaches: the BLRM coupled with the Bayesian LASSO and the BLRM coupled with the Spike and Slab prior.

#### 3.4.1 BLRM Coupled With the Bayesian LASSO (BLRM‐LASSO)

The LASSO method permits dealing with variable selection in a general regression setting. Where the number of covariates is considered to be high, the LASSO tends to ‘shrink’ some parameters to zero by adding a penalization parameter λ in the regression model, which permits both to reduce the variance of the least square estimator and to ease the interpretation of the regression [17]. The LASSO also has a Bayesian interpretation, since penalizing the likelihood function to estimate the parameters is equivalent to considering a prior distribution on the parameters' space [17]. The LASSO regression (with the mean square error approach) can be interpreted as a Bayesian estimation method through the mode of the posterior distribution, the prior being a Laplace distribution (also called double exponential):

(9) $$\pi (\gamma_{j})=\frac{\lambda_{p}}{2}\exp\left(-|\gamma_{j}|\lambda_{p}\right)$$

$\lambda_{p}$ being the penalization parameter. Several prior distributions are possible, on $\left(\lambda_{p}\right)$ [29, 30] or $\left(\lambda_{p}^{2}\right)$ [7, 18]. For the BLRM coupled with Bayesian LASSO (BLRM‐BLASSO), the probability of toxicity was also modeled with Equation (6). In that case, for intercept α and slope β the priors were the same as in the BLRM, while for each additional covariate's parameter $\gamma_{j}$ a hierarchical model was proposed, with one $\lambda_{p}$ per group of covariate depending on their distribution, with p=1,…,g being the index of the group:

(10) $$\left\{\begin{array}{l} \gamma_{j}|\lambda_{p}∼ℒaplace(0,\lambda_{p}),\;\text{for all covariates}\;X_{j}\;\text{in group}\;p\\ \lambda_{p}∼ℐnv𝒢amma(\delta ,\delta ) \end{array}\right.$$

δ being strictly positive. For instance, if $\gamma_{1},\ldots ,\gamma_{k}$ are the coefficients of continuous covariates and $\gamma_{k+1},\ldots ,\gamma_{k+l}$ the coefficients of binary covariates, then there are two kind of covariates (continuous and binary: g=2) and we considered one $\lambda_{1}$ and its prior distribution for the first k coefficients and one $\lambda_{2}$ with another prior distribution for the l binary covariates.

The dose‐allocation rule is the same as described for the BLRMc with covariate; just the prior distribution changed. This model always includes all covariates in the model and lets the estimate define the contribution to the dose‐toxicity‐covariate relationship.

#### 3.4.2 BLRM With Spike‐And‐Slab

Still with the same model for the probability of toxicity (6) and the same prior distribution for (α,log(β)), an other possibility for the additional covariates' parameter is to use the Spike‐and‐Slab

$$\left\{\begin{array}{l} \gamma_{j}|m_{j}∼(1-m_{j})f_{1}(\gamma_{j})+m_{j}f_{0}(\gamma_{j})\\ m_{j}∼ℬeta(b_{1},b_{2}) \end{array}\right.$$

with $m_{j}\in [0,1]$ being the mixture parameter and $f_{0}(\gamma_{j})$, $f_{1}$ being normal densities such that $f_{1}$ has a density with fewer variance than $f_{0}$. This model was called the BLRM‐2S. The dose‐allocation rule is the same as described for the BLRMc with covariate, just the prior distribution changed. All covariates are always included in this model, and then the estimate defines the contribution to the dose‐toxicity‐covariate relationship. This differs from the approach proposed Cotterill and Jaki [12] since here no specific subgroup additional term for the dose‐toxicity relationship is considered, and the Spike and Slab prior distribution are considered only for the covariates (not the dose effect related to a subgroup). The dose‐allocation rule is also the same as described for the BLRMc with covariate, and all covariates are included since the beginning of the trial in the model and let the estimate define the contribution to the dose‐toxicity‐covariate relationship.

## 4 Simulation Study

### 4.1 Settings

The performances of the proposed methods were evaluated through an extensive simulation study with different numbers of covariates (none, one, and two) having different distributions (continuous or discrete). The target toxicity rate is τ=0.25. The number of patients per trial was N=30, and patients were enrolled in a cohort of $n_{c}=3$. The first cohort of patients is allocated to the lowest dose $d_{1}$. The aim is to evaluate the performance of each method in comparison to a reference method, the BLRM, without dose personalization. Cases where one should and should not include covariates in the dose recommendation were considered. This allows us to investigate how the conventional BLRM performs when significant covariates are not taken into account and, secondly, to evaluate the performance of the models when non‐significant covariates are included in the model and explore how robust they are to this inclusion.

### 4.2 Data Generation

To generate the data, the two‐parameter logistic model was used for the dose‐toxicity relationship, with various numbers of covariates (no covariates, one, and two) and various distributions for the covariates, continuous and discrete. The discrete dose range was 𝒟=(1mg,3mg,5mg,7mg,9mg), and the reference dose was $d_{r}=5$. Under no covariates, the coefficients chosen for α and β (given in Table A.1 of Supporting Information) lead to the probability of toxicity per dose given in Table 1. Since no covariates are considered in that case, there was no pMTD but just *the* MTD. We considered five scenarios, one for each dose being the MTD.

**TABLE 1:** Probabilities of toxicity per dose when no covariates were used to generate the data.

Scenario	MTD	$p_{T}(d_{1})$	$p_{T}(d_{2})$	$p_{T}(d_{3})$	$p_{T}(d_{4})$	$p_{T}(d_{5})$
1	$d_{1}$	**0.25**	0.39	0.46	0.51	0.55
2	$d_{2}$	0.04	**0.24**	0.47	0.64	0.75
3	$d_{3}$	0.003	0.07	**0.24**	0.45	0.62
4	$d_{4}$	0.001	0.03	0.11	**0.25**	0.42
5	$d_{5}$	0	0.001	0.02	0.09	**0.26**

Continuous covariates are generated from a standard normal distribution 𝒩(0,1), assuming for the given setting (e.g., an actual trial) it would be possible to center and standardize the observed covariates using the available knowledge about their distribution, similarly to the motivating example, in which the distribution of methadone doses was known based on previous data. In the cases where covariates were used to generate the data, the probability of toxicity at a given dose level depends also on the value of these covariates. Therefore, we define the *average probability of toxicity* of a dose d as the marginal probability of toxicity: this is the average of probabilities of toxicity averaged across all possible values of covariates:

$$p_{T}(d)=\int_{𝒳}p_{T}(Y=1|X=x)q(x)dx$$

with q(·) being the distribution of the covariate X. To generate the data using covariates, the covariates were first generated, and then the patient response was generated from a Bernoulli distribution given these covariates.

The coefficients to generate the data were then chosen so that the average probability of toxicity matched the toxicity probabilities under the case with no covariates as in Table 1. This permits comparing the results of the BLRM without covariates to assess the impact of not including significant covariates. Under the cases with significant covariate(s), the probabilities given in Table 1 are not the pMTD but the *average probability of toxicity*. We again define five scenarios (denoted $d_{1}$, $d_{2}$, $d_{3}$, $d_{4}$, and $d_{5}$), and for each one an *average MTD* is the dose with the *average probability of toxicity* closest to the target rate τ=0.25 (the corresponding values for α, β, and the covariates coefficients $\gamma_{i}$ are given in Table A.1 of Supporting Information). When two covariates are considered for inclusion, they are not correlated.

**FIGURE 1:**
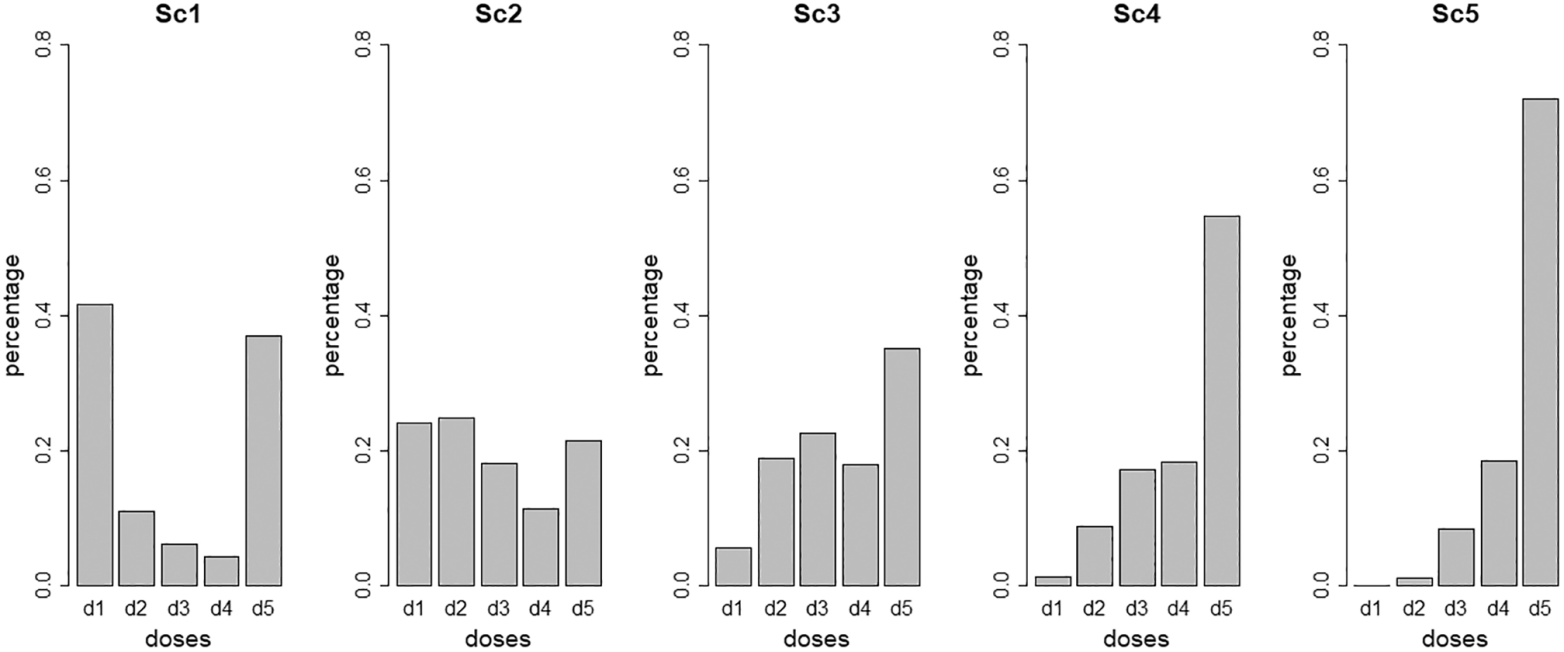
Repartition of the pMTD for the five scenarios when two normal covariates were used to generate the data. Each figure gives the percentage of patients having each dose as pMTD.

As an example, the pMTD distributions for each scenario with two significant normal covariates are given in Figure 1, and for each (*average MTD*) scenario, the percentage of the population having a certain dose as its pMTD. For instance, in Scenario 1, 41.9% of the population has dose $d_{1}$ as its pMTD, 10.9% dose $d_{2}$, 6.1% dose $d_{3}$, 4.3% dose $d_{4}$, and 36.9% dose $d_{5}$. Similarly, five scenarios were considered and constructed with one binary (generated with probability 0.5) covariate and one continuous covariate, and for each scenario, the corresponding distribution of the pMTD are given in the Supporting Information.

### 4.3 Performance Metrics

Several metrics were considered to evaluate the results. First, instead of having a single MTD at the end of the trial, a personalized pMTD should be considered. Therefore, the conventional dose‐finding metric, the proportion of *the* correct dose selection, might not be appropriate under all scenarios. To obtain an accuracy performance measure in terms of the pMTD recommendations and to take into account the distribution of the covariates in the population, at the end of each trial, 100 additional patients were simulated using the true dose‐toxicity relationship. For each of these new patients, their pMTD was calculated, and the average proportion of allocation to their pMTD (referred to as “MTD.add,” for “additional”) and the average proportion of overdosing (referred to as “Ov.add”) for each trial was calculated. Additionally, the operating characteristics of each method during the trial were also evaluated in terms of the percentage of patients allocated to their pMTD (MTD.trial) or overdosed during the trial (Ov.trial). For each of these four metrics, the average for the 1000 trials was considered. Note that the average values for MTD.add and MTD.trial are the geometric means [31], while we used the arithmetic mean for Ov.add and Ov.trial (the geometric one penalizing lower values [31], it is not adapted to evaluate the overdosing).

### 4.4 Prior Distribution

For the BLRM model, a bivariate normal non‐informative prior with a large variance was used [32]:

(11) $$\left(\begin{array}{l} \alpha \\ \log(\beta ) \end{array}\right)∼ℳ𝒩\left(\left[\begin{array}{l} \text{logit(0.1)}\\ 0 \end{array}\right],\left[\begin{array}{ll} 4 & 0\\ 0 & 4 \end{array}\right]\right)$$

For the BLRMc model, each parameter for the covariates had a flat normal prior $\gamma_{j}∼𝒩(0,4)$ with the same variance as the dose‐specific coefficient, since no penalization of coefficients was considered for the BLRMc. The choice of the parameters and hyperparameters for other parameters was done through a calibration.

#### 4.4.1 Calibration for Two Normal Covariates

In the case of the inclusion of two normal covariates, the hyperparameters for each coefficient $\gamma_{i}$ were chosen to be the same, so each covariates is treated similarly. This is because the distribution of the two covariates was the same (a normal distribution), and it was assumed that each of these covariates has the same influence on the dose‐toxicity relationship.

**FIGURE 2:**
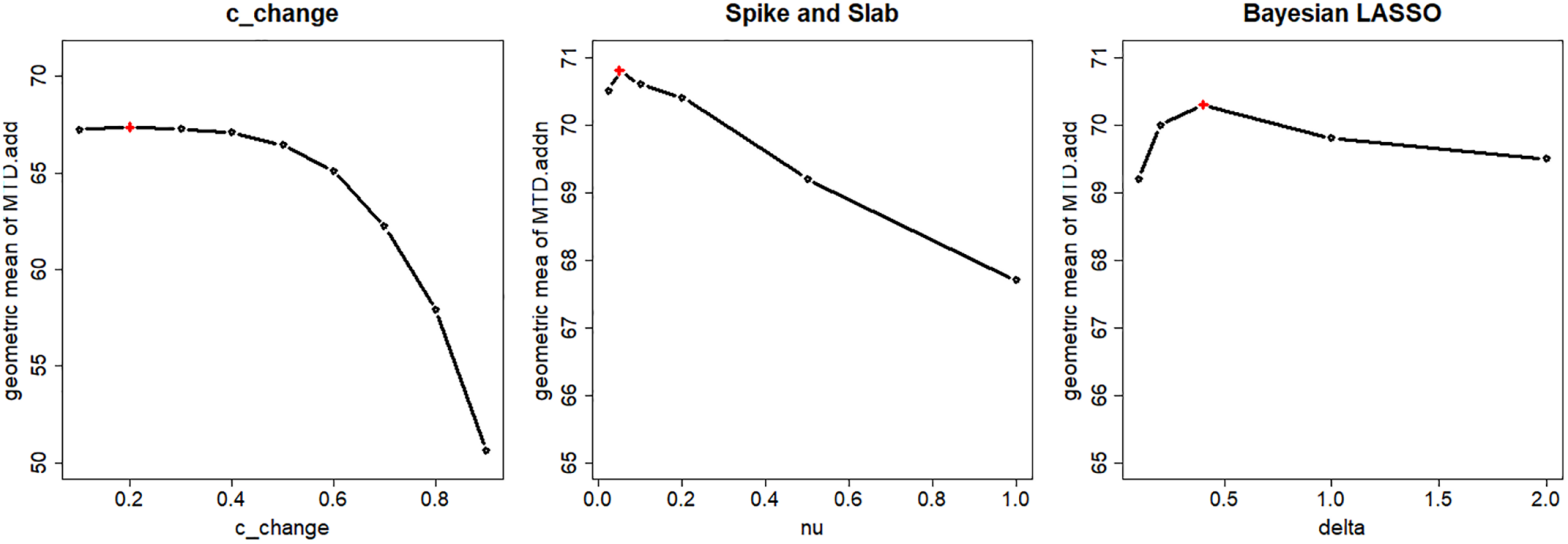
Calibration of the design/hyper‐prior parameter for $c_{change}$ (Left Panel), Spike&Slab (Middle Panel), and LASSO (Right Panel). The geometric mean of the average proportion of correct allocation for the additional patients (MTD.add) is calculated across all scenarios using 1000 simulations. The red cross in each plot indicates the chosen value.

For the $c_{change}$ criterion, several values were evaluated under all five considered scenarios of the *average MTD* location, and the geometric mean of the average proportion of correct allocations for the additional patients (MTD.add) for each value of $c_{change}$ was taken across all scenarios with none, one, or two covariates used to generate the data. The values tried for $c_{change}$ were from 0.1 to 0.9 by step 0.1. The values of the geometric mean of the average proportion of correct allocations for the additional patients ranged from 50.7 to 67.4, the optimal value 67.4 being obtained with $c_{change}=0.2$ (Left Panel of Figure 2).

For the Bayesian LASSO, the calibration was done on the hyperparameter δ (10). The smaller δ, the more the coefficient is penalized. The δ hyper‐parameter ranged from 0.1 to 2, and the optimal value for the geometric mean of pMTD allocation (ranged from 69.2 to 70.3) was δ=0.4 (see Right Panel of Figure 2).

For the Spike and Slab priors, the mixture parameter follows a Beta distribution $m_{j}∼ℬeta(2,2)$, the variance for the Slab was 4 (so the variance of the flat component of the prior matches the variance of the BLRMc approach), while a calibration was done to find the variance for the Spike's variance ν (since it is the hyper‐parameter that controls the penalization of the covariate's coefficient): several values (0.025, 0.05, 0.1, 0.3, and 0.5) were investigated; the geometric mean of correct pMTD allocations ranged from 67.7 to 70.8, the optimal value obtained with a Spike's variance equals to ν=0.05 (see Middle Panel of Figure 2).

#### 4.4.2 Calibration for One Binary and One Normal Covariate

We also performed a prior calibration for the case of two mixed covariates: one binary and one normal, and evaluated several combinations for the hyperparameters for the covariate's coefficient's prior. We allow for possibly different values of the hyperparameters to account for the different nature of the covariates, continuous or discrete. The values evaluated were the optimal ones in the sequence of evaluated parameters for the two normal covariates and the immediate previous and following ones, giving a total of nine combinations to evaluate. All detailed results are available in Supporting Information (Table A.9 for the $c_{change}$ criterion, Table A.10 for the LASSO and A.11 for Spike and Slab). For the $c_{change}$ criterion, the best combination in terms of pMTD allocation is $c_{change}=0.1$ for the normal covariate and $c_{change}=0.3$ for the Bernoulli covariate, while for the Bayesian LASSO, two different prior distributions were used for the continuous and binary covariates, and δ=1 for the normal covariate and δ=0.4 for the Bernoulli covariate was the optimal combination. For the Spike variance, the best combination in terms of pMTD allocation is ν=0.1 for the normal covariate and ν=0.05 for the Bernoulli one.

Computations were carried out in R (version 3.3.3), and rstan package (version 2.21.2) was used for Bayesian inference.

## 5 Results

### 5.1 Case of Two Normal Covariates Included

It is expected that when no covariate had an influence on the dose‐toxicity relationship, the BLRM should give the optimal results (in terms of pMTD allocation during the trial and for additional patients, as well as for the operational characteristics during the trial), and when one or two covariates were used to generate the data, the BLRMc including one and two covariates, respectively, should perform better than the other models in terms of the considered metrics. Similarly, when no covariates had an influence on the dose‐toxicity relationship, we expect that the BLRMc, since it does not consider any penalization, should give the worst results in terms of operational characteristics and pMTD allocation. For this reason, we presented the results by the number of covariates used to generate the data.

In Table 2 the results for the inclusion of two continuous covariates are presented. In Table 2, the metrics MTD.add and Ov.add are given considering the three cases where the data were generated without any continuous covariates, one continuous, and two continuous, while the MTD.trial and Ov.trial are given in Table A.3 of Supporting Information.

**TABLE 2:** Average proportion of patients (out of 100) assigned to their pMTD at the end of the trial and to an overdose for inclusion of two continuous covariates under different scenarios ($d_{1}$ to $d_{5}$) to simulate the data with none (so only intercept and slope in the model), one or two continuous covariates. Results are based on 1000 simulations.

		MTD.add						Ov.add					
Covariates	Model	d1	d2	d3	d4	d5	Mean	d1	d2	d3	d4	d5	Mean
0	BLRM	70	80	78	49	83	71	30	14	12	20	0	15
	BLRMc	59	61	60	34	68	55	41	24	18	25	0	22
	$c_{change}$	60	62	59	36	72	56	40	23	20	26	0	22
	Spike Slab	69	73	70	46	76	66	31	17	16	22	0	17
	Lasso	68	74	71	47	78	67	32	17	16	23	0	18
1	BLRM	33	24	23	27	60	31	13	27	25	25	22	22
	BLRMc1	82	78	77	78	80	79	11	13	12	10	8	11
	BLRMc2	79	73	70	70	74	73	12	15	13	12	10	12
	$c_{change}$	80	73	71	72	75	74	11	15	14	11	10	12
	Spike Slab	82	78	75	74	76	77	12	13	11	10	11	11
	Lasso	79	73	69	70	75	73	12	14	12	14	13	13
2	BLRM	31	24	22	26	58	30	15	27	26	26	23	23
	BLRMc2	79	73	71	71	74	74	12	15	13	12	10	12
	$c_{change}$	79	73	72	71	73	73	12	15	13	12	12	13
	Spike Slab	78	71	67	65	71	70	13	15	13	14	14	14
	Lasso	78	70	68	68	74	71	12	15	13	15	14	14

#### 5.1.1 No Covariates to Generate the Data

The BLRM outperforms the other methods when no covariates have an impact on the dose‐toxicity relationship: the geometric mean of the percentage of correct pMTD allocation for the additional patients across the five scenarios (71%) is the highest. Spike and Slab and LASSO result in the second highest averages (66% and 67% correct pMTD allocation on average, with similar results to the BLRM for scenarios $d_{1}$ and $d_{4}$), while the BLRMc and $c_{change}$ give the lowest average performance, lower than the BLRM by around 10% (55% and 56% correct pMTD allocation). The overdosing for additional patients is the lowest with the BLRM (15%) but comparable with Spike and Slab and LASSO (17% and 18%), while the BLRMc and $c_{change}$ have the highest overdosing (22%), except for scenario $d_{5}$, where no overdosing is possible.

The BLRM also outperforms the other methods in terms of operating characteristics during the trial (see corresponding results in Table A.3 of Supporting Information): the percentage of correct pMTD allocation is the highest (48%), while the overdosing is the lowest (21%). Its performance is better than all other methods for all five scenarios considered for the pMTD allocation during the trial, the lowest average being obtained by BLRMc with covariates (38% vs. 48% for the BLRM and 40% for $c_{change}$).

#### 5.1.2 One Normal Covariate Used to Generate the Data

In the setting where one normal covariate is used to generate the data, it is expected that the BLRMc1 (with one covariate included only) will result in the best performance in terms of considered operating characteristics. For the percentage of correct pMTD allocations for the additional patients, the geometric mean (79%) is the highest among all methods, followed by Spike and Slab (77%), while BLRMc2, LASSO, and $c_{change}$ result in similar average proportions (73% and 74% on average, with maximum difference with BLRMc1 under scenario $d_{3}$, 8% lower for LASSO). BLRMc1 has same performance as the Spike and Slab for scenarios $d_{1}$ and $d_{2}$, but outperforms all other models under other scenarios. The overdosing for the additional patients is the same for the BLRMc1 and the Spike and Slab on average and lower than other methods. The BLRMc1 also shows better performance in terms of correct pMTD allocation during the trial (see Table A.3 of Supporting Information). The BLRM (without covariates) shows the worst performance in term of pMTD allocation for additional patients (31%: 40% less than other methods) and overdosing (22%, so more than 9% than other methods), as well as for the operational characteristics during the trial.

#### 5.1.3 Two Normal Covariates Used to Generate the Data

In the setting where two covariates have an influence on the dose‐toxicity relationship, the BLRMc2 with two covariates results in the best performance for the additional patients' percentage of correct pMTD allocations among all models: the geometric mean (74%) is the highest. The $c_{change}$ criterion gives the same results for scenarios $d_{1}$, $d_{2}$, and $d_{4}$ (respectively 79%, 73%, and 71%) and has an average of 73%. Spike and Slab and LASSO have an average of correct pMTD allocation of 70% and 71%, up to 6% of difference with BLRMc2. The overdosing for the additional patients on average is the lowest for BLRMc2 (12%), being lower by 1% than using $c_{change}$ model but the same for scenarios $d_{1}$, $d_{2}$, $d_{3}$, and $d_{4}$ (12%, 15%, 13%, and 12%). All methods give the same results for scenarios $d_{2}$ and $d_{3}$ (15% and 13%), except the BLRM without covariates (27% and 26%), which gives the lowest results for all metrics.

Overall, the case of two covariates inclusion shows that, on average, it is beneficial to include covariates but also to consider a model with penalization. Indeed, including covariates when it is not necessary leads to 6% to 16% lower, on average, proportion of correct allocations compared to the BLRM. The penalization methods can decrease this to 2%–6%, while the $c_{change}$ method in this setting gives similar results to including covariates. On the other hand, the losses in the operating characteristics if the covariates should be included but they are not (i.e., by the BLRM) can be significantly higher. Specifically, the BLRM will result in 44%–48% lower proportion of correct allocations at the end of the trial (compared to the reference method with the correct number of covariates). At the same time, the penalization methods result in very minor decreases in the operational characteristics compared to the reference methods.

Similarly, including covariates when no covariates were used to generate the data leads to an increase of overdosing for additional patients in comparison to the BLRM, but this overdosing is lower on average when using penalization methods such as LASSO and Spike and Slab than the BLRMc (and $c_{change}$, which gives similar results to the BLRMc). Otherwise, the penalization methods lead to similar overdosing to the BLRMc when one or two covariates were used to generate the data (with never more than 5% of difference with the BLRMc scenario by scenario), while the BLRM leads to an overdosing higher by at least 10% than the BLRMc.

### 5.2 Case of One Binary and One Normal Covariate Included

In Table 3, the results for the inclusion of two continuous covariates are presented. In Table 3, the metrics MTD.add and Ov.add are given considering the four cases where the data were generated without any covariates, one continuous, one binary, and two covariates (one normal and one Bernoulli), while the MTD.trial and Ov.trial are given in Table A.5 of Supporting Information.

**TABLE 3:** Average proportion of patients (out of 100) assigned to their pMTD at the end of the trial and to an overdose for inclusion of two covariates (one continuous and one binary) under different scenarios to simulate the data, with no covariate to generate the data, one binary covariate to generate the data (1b), one continuous covariate to generate the data (1c), or two covariates (one continuous and one binary) to generate the data (2mix).

		MTD.add						Ov.add					
Covariates	Model	d1	d2	d3	d4	d5	Mean	d1	d2	d3	d4	d5	Mean
0	BLRM	70	80	78	49	83	71	30	14	12	20	0	15
	BLRMc	62	63	61	37	67	57	38	23	19	23	0	21
	$c_{change}$	62	63	61	38	68	57	38	24	19	24	0	21
	Spike Slab	68	72	71	46	71	65	32	18	14	20	0	17
	Lasso	68	72	70	47	74	65	32	19	16	22	0	18
1b	BLRM	48	48	12	27	77	36	16	8	44	36	0	21
	BLRMc1	64	56	45	52	70	57	21	12	38	23	0	19
	BLRMc2	56	53	34	48	70	51	29	19	41	24	0	23
	$c_{change}$	59	52	44	46	69	53	24	15	36	24	0	20
	Spike Slab	59	49	44	42	72	52	19	15	35	27	0	19
	Lasso	58	54	39	42	73	52	18	13	38	29	0	20
1c	BLRM	32	24	22	27	59	31	13	27	25	25	21	22
	BLRMc1	82	78	78	78	80	79	11	13	12	11	8	11
	BLRMc2	80	74	73	74	77	75	12	15	13	12	9	12
	$c_{change}$	80	74	74	74	76	76	12	15	12	11	9	12
	Spike Slab	81	78	76	74	76	77	12	12	10	11	10	11
	Lasso	81	76	74	74	78	76	12	13	12	12	11	12
2mix	BLRM	41	42	26	27	57	37	17	24	26	25	22	23
	BLRMc2	64	58	62	63	71	64	20	21	15	14	11	16
	$c_{change}$	64	58	62	62	70	63	20	21	15	15	12	17
	Spike Slab	59	52	55	58	70	58	20	22	17	17	14	18
	Lasso	59	53	53	58	71	59	19	22	19	18	15	19

#### 5.2.1 No Covariates to Generate the Data

In the setting where no covariate has an influence on the dose‐toxicity relationship, the BLRM outperforms all methods on average and scenario by scenario. In terms of pMTD allocation rate for additional patients, it outperforms the BLRMc2 and $c_{change}$ by 14% (71% vs. 57%) with a maximum of 17% of differences for scenarios $d_{2}$ (63% vs. 80%) and $d_{3}$ (61% vs. 78%) and is 6% better on average than Spike and Slab and LASSO (both 65% with a maximum of 12% of difference in scenario $d_{5}$). The overdosing for additional patients is the lowest (15%) but similar to Spike and Slab (17%) with a maximum of 4% of difference for scenario $d_{2}$ (14% vs. 18% for Spike and Slab).

#### 5.2.2 One Binary Covariate to Generate the Data

In the setting where one binary covariate has an impact on the dose‐toxicity relationship, the BLRMc1 outperforms all methods in terms of pMTD allocations for the additional patients, with an average of 57%, followed just after by the $c_{change}$ with an average of 53% and a maximum difference of 6% in scenario $d_{4}$ (46% vs. 52% for BLRMc1). BLRMc2, Spike and Slab, and LASSO show similar performance (51% and 52% on average). The worst performance in terms of pMTD allocations is obtained with the BLRM (36%). The overdosing for the additional patients is the lowest withe Spike and Slab model (both 19% on average) but with differences scenario by scenario (better in scenario $d_{4}$: 23% vs. 27%, but worse in scenario $d_{3}$, for instance: 38% vs. 35%).

#### 5.2.3 One Normal Covariate to Generate the Data

In the setting where one continuous covariate was used to generate the data, the BLRMc1 outperforms all methods in terms of pMTD allocations for additional patients (79%) and has comparable results to Spike and Slab (77%) with never more than 4 of difference scenario by scenario, while BLRMc2, $c_{change}$, and LASSO show similar results and have never more than 5 of difference with the BLRMc1 scenario by scenario. The overdosing for all methods (except BLRM is similar, as well as the pMTD allocations during the trial and the overdosing during the trial: BLRMc1 shows the best performance, but scenario by scenario the difference is never more than 4%. The BLRM gives the worst results in terms of pMTD allocations for additional patients (31%) and overdosing (22% vs. 11% and 12% for other methods).

#### 5.2.4 One Normal and One Binary Covariate to Generate the Data

In the setting where the two covariates were used to generate the data, the BLRMc2 outperforms all methods on average of pMTD rate for additional patients (64%), but $c_{change}$ shows similar results (63%) with never more than 1% of difference scenario by scenario, as well as for the overdosing for additional patients (16% and 17%), and same average of pMTD allocations during the trial (42% with never more than 2% of difference) and similar overdosing during the trial. BLRMc1 has on average 5%–6% more good pMTD allocations for additional patients than other methods (except BLRM: 37% on average) with a maximum of 9% of difference in scenario $d_{3}$ with LASSO (53% vs. 62% for BLRMc2). There is never more than 4% difference scenario by scenario for the overdosing of additional patients, except for BLRM (23% on average against 16% for BLRMc2 and 17%, 18%, and 19% for $c_{change}$, Spike and Slab, and LASSO).

Overall, the case of two covariates inclusions (one binary and one continuous) shows that on average, it is beneficial to include covariates, and also to consider a model with penalization. Including covariates when it is not necessary leads to a 14% lower, on average, proportion of correct allocations compared to the BLRM. The penalization methods can decrease this to 6%, while the $c_{change}$ method in this setting also gives similar results to including covariates (BLRMc). On the other hand, the losses in the operating characteristics if the covariates should be included but they are not (i.e., by the BLRM) can be significantly higher. Specifically, the BLRM will result in 20%–48% lower proportion of correct allocations at the end of the trial (compared to the reference method with the correct number of covariates). At the same time, the penalization methods result in very minor decreases in the operational characteristics compared to the reference methods (never more than 6% on average).

Similarly, including covariates when no covariates were used to generate the data leads to an increase of overdosing for additional patients in comparison to the BLRM (6% higher with BLRMc and $c_{change}$), but this overdosing is lower on average when using penalization methods such as LASSO and Spike and Slab than the BLRMc (and $c_{change}$, which gives similar results to the BLRMc). Otherwise, the penalization methods lead to similar overdosing to the BLRMc when one or two covariates were used to generate the data (with never more than 5% of difference with the BLRMc scenario by scenario), while the BLRM leads to a higher overdosing.

## 6 Two Normal Correlated Covariates

Some covariate, such as biomarkers, can be highly correlated [6], therefore, the case of correlated covariates was also investigated (including negative and positive correlations). The results for additional patients for several correlations between two normal covariates used to generate the data are presented in Table 5, and in Table A.7 of Supporting Information are the results for operational characteristics during the trial. BLRMc2, BLRMc1, LASSO, and Spike and Slab were investigated. The BLRMc2 was investigated because we expect this method to give the optimal results in terms of operational characteristics for the same reasons as before, the BLRMc1, to evaluate its performance, including only one covariate but correlated to the one missing, the Bayesian LASSO, and Spike and Slab to evaluate how penalization methods perform in presence of correlated covariates, since there is evidence in the literature that in some specific cases their performance (for instance of the LASSO) can be affected [33, 34].

The BLRMc2, which results in the best results in terms of considered operational characteristics, results in an average of correct pMTD allocations of 63% for correlation −0.8, 68% for correlation −0.5, and this average keeps increasing as the correlation increases, with 75% for correlation 0.8. The overdosing of BLRMc2, otherwise, decreases slightly as the correlation increases (from 15% for correlation −0.8 to 11% for correlation 0.95). The Spike and Slab and LASSO give similar results (never more than 2% of difference on average and 4% scenario by scenario) and also tend to improve their performance in terms of pMTD correct allocations as the correlation increases, from 61% and 59% for correlation −0.8 to 76% for correlation 0.95, and the overdosing for additional patients also decreases as the correlation increases (from 16% and 17% for correlation −0.8 to 12% for correlation 0.95). Finally, the BLRMc1 results in an improving performance in terms of considered metrics as the correlation increases (pMTD correct allocations from 35% for correlation −0.8 to 74% for correlation 0.95, and overdosing from 22 to 13), while its results are lower optimal than the other methods.

We can note that the higher the correlation, the more comparable the results of the BLRMc1 to BLRMc2, although they are always lower in terms of pMTD correct allocations for additional patients. Additionally, the other methods give lower but comparable results to the BLRMc2, which indicates that considering the inclusion of correlated covariates using penalization methods, as in the case of no correlated covariates, permits to have similar operational characteristics to the BLRMc2.

## 7 Sensitivity Analysis

We also evaluated the operational characteristics of our methods when the data were generated by another model than the two‐parameter logistic model. We used the same dose range, and the covariates were generated from one or two standard normal distributions 𝒩(0,1). We generated the data from the Emax model [35]. Without covariates, the model is:

(12) $$\log\left(\frac{p_{T}(d_{k}|X_{i•})}{1-p_{T}(d_{k}|X_{i•})}\right)=\alpha_{1}+\frac{\alpha_{2}d_{k}}{d_{k}+\alpha_{3}}$$

while with covariates, the model is:

(13) $$\log\left(\frac{p_{T}(d_{k}|X_{i•})}{1-p_{T}(d_{k}|X_{i•})}\right)=\alpha_{1}+\frac{\alpha_{2}d_{k}}{d_{k}+\alpha_{3}}+\overset{p}{\underset{j=1}{\sum }}\gamma_{j}x_{ij}$$

We considered five scenarios, one for each dose being the MTD, in a similar way to in Section 4.2. The coefficients to generate the data were then chosen so that the average probability of toxicity matched the toxicity probabilities under the case with no covariates, as in Table A.13 in the Supporting Information.

### 7.1 No Covariates to Generate the Data

The BLRM outperforms the other methods when no covariates have an impact on the dose‐toxicity relationship: the geometric mean of the percentage of correct pMTD allocation for the additional patients across the five scenarios (53%) is the highest. LASSO, Spike and Slab, and change have similar results (50%, 48%, and 47% correct pMTD allocation on average, with similar results to the BLRM for scenarios $d_{1}$, $d_{4}$, and $d_{5}$), while the BLRMc gives the lowest average performance (41% correct pMTD allocation). The overdosing for additional patients is the lowest with the BLRM (20%) but comparable with Spike and Slab and LASSO (22%), while the BLRMc and $c_{change}$ have the highest overdosing (22%), except for scenario $d_{5}$, where no overdosing is possible.

The BLRM also outperforms the other methods in terms of operating characteristics during the trial (see corresponding results in Table A.15 of Supporting Information).

### 7.2 One Normal Covariate to Generate the Data

In the setting where one normal covariate is used to generate the data, it is expected that the BLRMc1 (with one covariate included only) will result in the best performance in terms of considered operating characteristics. For the percentage of correct pMTD allocations for the additional patients, the geometric mean (61%) is the highest among all methods, followed by Spike and Slab and BLRMc2 (55%), LASSO (53%) and $c_{change}$ (48%). The overdosing for the additional patients is comparable for BLRMc1, BLRMc2, Spike and Slab and LASSO, while the BLRM shows an overdosing at 25% on average. The BLRMc1 also shows better performance in term of correct pMTD allocation during the trial (see Table A.15 of Supporting Information). The BLRM (without covariates) shows the worst performance in term of pMTD allocation for additional patients (35%: 23% less than other methods).

### 7.3 Two Normal Covariates to Generate the Data

In the setting where two normal covariates are used to generate the data, it is expected that the BLRMc2 (with two covariates included) will result in the best performance in terms of considered operating characteristics. For the percentage of correct pMTD allocations for the additional patients, the geometric mean (59%) is the highest among all methods, followed by Spike and Slab, and LASSO (55% and 53%) and $c_{change}$ (48%). The overdosing for the additional patients is comparable for BLRMc2, Spike and Slab and LASSO, while the BLRM shows an overdosing at 25% on average. The BLRMc2 also shows better performance in terms of correct pMTD allocation during the trial (see Table A.15 of Supporting Information). The BLRM (without covariates) shows the worst performance in terms of pMTD allocation for additional patients (33%: 25% less than other methods).

Overall, including covariates considering penalization methods permits a good trade‐off between not including a significant covariate and the risk of including a non‐significant covariate. If a non‐significative covariate is included considering penalization, the correct pMTD selection at the end of the trial or pMTD allocation during the trial can decrease, but on average we don't lose more than 5%–6% (but it can be more scenario by scenario). However, not including any significant covariate decreases the correct pMTD selection at the end of the trial by at least 20%, while the overdosing during the trial (and at the end of the trial in terms of pMTD allocation) is higher with the BLRM but comparable to other methods. The results are given in (Table 4).

**TABLE 4:** Average proportion of patients (out of 100) assigned to their pMTD at the end of the trial and to an overdose for inclusion of two continuous covariates under different scenarios ($d_{1}$ to $d_{5}$) to simulate the data with none (so only $\alpha_{1}$, $\alpha_{2}$, and $\alpha_{3}$ in the Emax model), one or two continuous covariates. Results are based on 1000 simulations.

		MTD.add						Ov.add					
Covariates	Model	d1	d2	d3	d4	d5	Mean	d1	d2	d3	d4	d5	Mean
0	BLRM	86	67	49	26	58	53	14	26	31	32	0	20
	BLRMc	72	50	36	20	47	41	28	33	32	29	0	24
	$c_{change}$	81	59	41	23	58	48	19	30	35	35	0	24
	Spike Slab	83	61	39	24	52	47	17	27	34	33	0	22
	Lasso	83	62	40	26	56	50	17	28	34	33	0	22
1	BLRM	66	35	18	30	41	35	8	29	32	30	27	25
	BLRMc1	71	56	63	55	61	61	20	26	19	17	13	19
	BLRMc2	67	50	57	49	54	55	24	28	19	17	14	20
	$c_{change}$	65	42	46	41	50	48	15	31	24	24	21	23
	Spike Slab	68	48	57	48	55	55	16	28	20	21	17	20
	Lasso	69	45	52	47	55	53	15	28	23	23	20	22
2	BLRM	64	28	19	28	42	33	7	29	33	30	27	25
	BLRMc2	68	54	54	57	62	59	22	25	21	16	13	20
	$c_{change}$	64	40	40	45	55	48	13	30	28	24	21	23
	Spike Slab	66	46	47	51	58	53	16	26	25	21	18	21
	Lasso	68	48	47	52	60	55	14	25	25	22	19	21

### 7.4 Blrmc With Higher Sample Sizes

**FIGURE 3:**
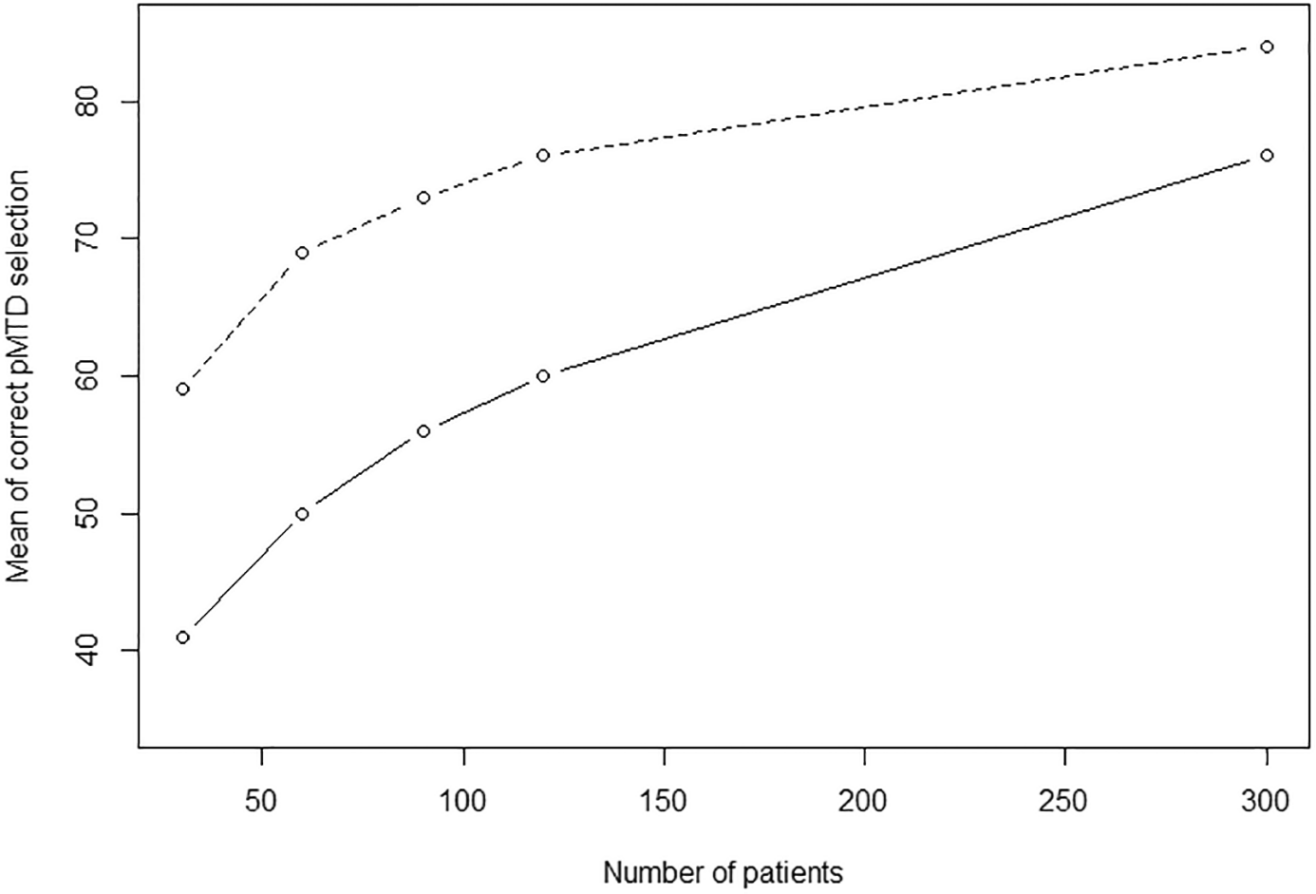
Geometric mean of the percentage of correct pMTD selection at the end of the trial across the five scenarios for trials of 30, 60, 90, 12, and 300 patients. The solid line is for the case where the data were generated without covariates with the Emax model and two normal covariates were included, while the dashed line is for the case where the data were generated with the Emax model with two normal covariates (included also in the model). Results are based on 1000 simulations.

The BLRMc2 with covariates increases the number of parameters of the model, and then the question of convergence can be raised [36]. We evaluated the convergence of the BLRMc2 with covariates in the setting where the data were generated with the Emax model, with zero or two normal covariates to generate the data. The results (see Figure 3) show that the BLRMc2 performance in terms of correct pMTD selection at the end of the trial increases with number of patients. In particular, the convergence is faster when the covariates included have an impact on the dose‐toxicity relationship, as expected, but remains at 80% or below of correct pMTD selection for 300 patients.

## 8 Discussion

We presented here several methods to deal with covariate inclusion in the dose‐finding framework in order to be able to allocate the correct pMTD to patients according to their individual covariates in a setting with a limited sample size. Those methods include an extension of the BLRM that includes covariates and Bayesian penalization (LASSO) and selection (Spike and Slab) methods. In this study we considered the cases of normal covariates, Bernoulli covariates, and mixtures of both. We explored the cases of independent and correlated covariates; the case of covariates with interaction could also be explored. Otherwise, all scenarios, in the several settings, were chosen to have the same average probability of toxicity per dose: scenarios in which this hypothesis is dropped could lead to different results. The simulation results show that considering inclusion or penalization methods when the covariates is not significant decreases the loss of correct pMTD allocation in comparison to not considering penalisation. Similarly, not including the covariates when they are significant leads to a drastic loss of a correct pMTD allocation, since the BLRM recommends only one unique dose, while penalization methods give results closer to the BLRMc with the correct number of significant covariates included.

Considering penalization methods is therefore a good trade‐off between completely omitting the covariates (BLRM) and including them without penalization (BLRMc). Those methods lead to good operational characteristics in term of pMTD allocation during the trial and in terms of dose recommendation for additional patients, as well as in terms of overdosing of additional patients and overdosing during the trial.

Except for the $c_{change}$ method, in which the covariates can be included during the trial if the criterion is reached, in the other methods we considered, the covariates were always included at the beginning of the trial, and to maintain clarity for the interpretation, it was not possible to exclude a covariate. However, it remains possible to assess at the end of the trial if a covariate has an influence on the dose recommendation, since a too low coefficient will not lead to a modification of the recommendation of the dose. In this way, some extensions of the proposed methods can be proposed with covariate selection at the end of the trial, considering their posterior distribution (as investigated by Kakurai et al. [7]) or their influence in term of dose allocation (like the $c_{change}$ criterion). The $c_{change}$ criterion, otherwise, showed it tended to include the covariates even when the covariate were not significant (see Tables A.12 and A.13 in Supporting Information).

All prior distributions for the covariates have a variance of 4. In practice, some covariates can have very different scales. We have chosen the case of normal and binary covariates. To address this issue, a solution could be to center and standardize the covariates to be considered for inclusion during the trial, which would make them comparable in terms of variance.

Including more than one or two covariates could be considered. The question of the inclusion of covariates in a Phase I trial requires a good rationale on why each of these should be included in the model and why it is believed to affect the drivers of toxicity. The decision to include covariates should have a strong clinical justification. We have limited our consideration to 1 and 2 covariates due to these options being discussed in our motivating trial. In practice, our suggestion is that, for a particular setting with more than 2 covariates to be included, a sensitivity analysis should be performed depending on the number and nature (continuous or discrete) of the covariates.

While the motivating case study considered the setting of the dose‐finding trial in opioid detoxification, the considered methodology can be applied in dose‐finding trials in patients in other therapeutic areas, for example, in oncology dose‐finding trials. Furthermore, the considered setting does not include the placebo arm as it concerns modeling the risk of event of the experimental drug. The placebo arm, however, can be added as dose zero in the model supported by the objectives of the trial.

Finally, our work focused on phase I dose‐finding trials, considering the influence of covariates on dose‐toxicity relationships. In the motivating trial, the covariates being discussed were not limited to the binary or ordinal covariates with a clear assumption on what groups will have a higher risk of toxicity a priori, hence, we have approached under a general framework on adding covariates into the trial/model. However, in some settings, the setting of adding covariates can be suitable (with ordering [37] or population heterogeneity, for [38]). An extension to phase I‐II with both dose‐toxicity and dose‐efficacy relationships could be explored, with the possibility of some covariates to have an influence on one relationship only or both.

**TABLE 5:** Average proportion of patients (out of 100) assigned to their pMTD at the end of the trial and to an overdose for inclusion of two correlated normal covariates under different scenarios to simulate the data. Several correlations, negative and positive, were considered.

		MTD.add						Ov.add					
Correlation	Model	d1	d2	d3	d4	d5	Mean	d1	d2	d3	d4	d5	Mean
−0.8	BLRMc2	72	57	58	61	69	63	12	19	17	15	12	15
	Spike Slab	73	53	58	56	67	61	14	20	15	16	15	16
	LASSO	70	50	54	54	69	59	13	21	17	18	16	17
	BLRMc1	33	32	26	31	60	35	18	25	24	23	18	22
−0.5	BLRMc2	74	61	68	64	72	68	14	19	15	14	10	14
	Spike Slab	70	54	68	57	69	63	15	21	13	16	14	16
	LASSO	70	54	66	57	71	63	14	20	13	17	15	16
	BLRMc1	42	33	27	36	63	39	17	25	25	23	17	21
−0.2	BLRMc2	77	58	70	74	72	70	13	22	13	13	10	14
	Spike Slab	75	50	66	70	70	65	14	24	12	12	14	15
	LASSO	75	52	66	71	72	67	13	22	13	13	14	15
	BLRMc1	46	43	33	36	66	43	16	25	24	24	16	21
0.2	BLRMc2	79	59	71	67	73	70	12	22	13	12	10	14
	Spike Slab	77	52	66	61	72	65	14	23	13	15	13	16
	LASSO	78	54	68	65	74	67	13	21	13	15	14	15
	BLRMc1	55	44	41	51	69	51	15	24	22	20	14	19
0.5	BLRMc2	79	63	73	71	73	72	12	20	13	12	10	13
	Spike Slab	77	56	67	67	72	67	13	22	14	14	13	15
	LASSO	78	57	70	69	74	69	12	20	13	14	14	15
	BLRMc1	64	50	50	60	71	58	14	23	20	17	13	17
0.8	BLRMc2	81	77	75	73	72	75	12	14	12	11	9	12
	Spike Slab	78	73	71	71	72	73	13	15	13	13	12	13
	LASSO	80	74	73	71	74	74	12	14	13	13	13	13
	BLRMc1	72	62	62	65	73	67	13	18	17	15	11	15
0.95	BLRMc2	80	79	77	74	73	77	13	13	11	11	9	11
	Spike Slab	80	78	76	74	73	76	13	13	11	11	11	12
	LASSO	80	78	75	73	75	76	12	13	12	12	12	12
	BLRMc1	77	73	73	73	75	74	13	15	14	13	9	13

## Conflicts of Interest

The authors declare no conflicts of interest.

## Supporting information




**Data S1** Supporting Information.
