# Supplementary material for: On Inclusion of Covariates in Model Based Dose Finding Clinical Trial Designs
Source: Stat Med. 2025 Jan 24;44(3-4):e10337. doi: 10.1002/sim.10337 (PMC11758501; doi:10.1002/sim.10337)
Supplement: Supplementary file 1 — Data S1 Supporting Information. [file SIM-44-0-s001.pdf]

Supplementary material for Article "On inclusion of covariates in model based dose finding clinical trial designs"

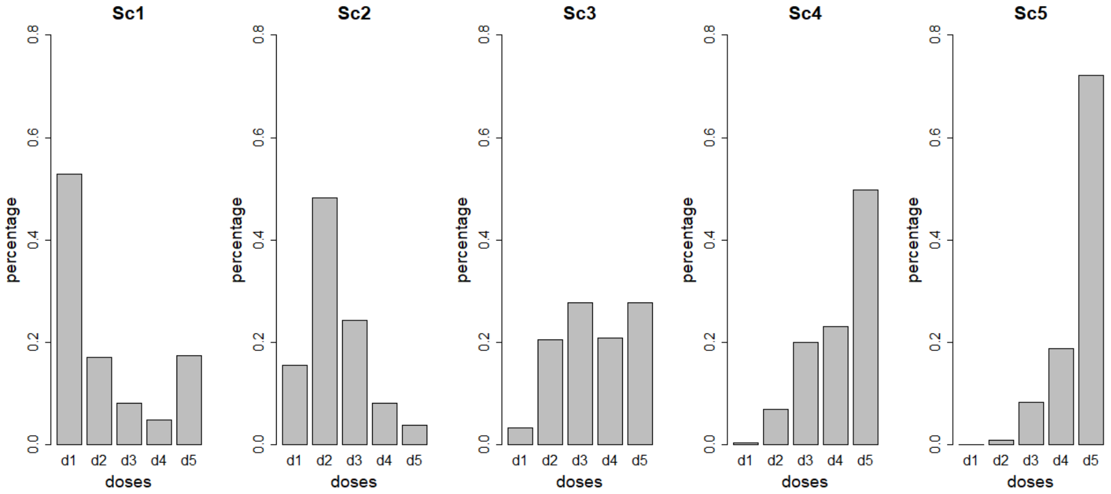

Figure 1: Repartition of the pMTD for the five scenarios when two covariates where used to generate the data: one normal and one Bernouilli. Each figure (one per Scenario) gives the percentage of patients having each dose as pMTD.

1 Additional Tables for the results section

1.1 Case of two normal covariates included

Average MTD	Covariates	α	β	γ_1	γ_2
	0	-0.14	0.60	0.00	0.00
	1c	-0.50	1.50	4.00	0.00
d_1	1b	-0.70	0.60	1.10	0.00
	2n	-0.50	1.50	3.00	2.50
	2mix	-0.80	-0.80	1.20	1.20
	0	-0.10	2.00	0.00	0.00
	1c	-0.30	5.30	4.10	0.00
d_2	1b	-1.40	2.60	2.50	0.00
	2n	-0.30	5.30	3.00	3.00
	2mix	-0.70	2.30	1.00	1.00
	0	-1.15	2.90	0.00	0.00
	1c	-3.00	7.20	4.00	0.00
d_3	1b	-2.80	3.70	2.60	0.00
	2n	-3.00	7.20	3.00	3.00
	2mix	-3.00	1.70	2.10	2.10
	0	-2.10	3.00	0.00	0.00
	1c	-4.50	6.20	3.20	0.00
d_4	1b	-3.00	3.00	1.50	0.00
	2n	-4.50	6.20	2.40	2.40
	2mix	-4.50	5.00	2.00	2.00
	0	-4.00	5.00	0.00	0.00
	1c	-5.00	5.50	2.00	0.00
d_5	1b	-3.50	3.30	0.80	0.00
	2n	-5.00	5.50	1.50	1.50
	2mix	-5.00	4.50	1.50	1.50

Table A.1: Values of each coefficient used to generate the data, without covariate (only α and β), one normal covariate (denoted 1c, with γ_1), one binary covariate (denoted 1b, with γ_1), two normal covariates (denoted 2n with γ_1 and γ_2) and two covariates (one normal and one binary, denoted 2mix with γ_1 and γ_2), for the five scenarios.

Covariates	Model	MTD.add						Ov.add					
		d1	d2	d3	d4	d5	Mean	d1	d2	d3	d4	d5	Mean
0	BLRM	70(46)	80(40)	78(41)	49(50)	83(38)	71	30(30)	14(14)	12(12)	20(20)	0(0)	15
	BLRMc	59(30)	61(24)	60(26)	34(26)	68(28)	55	41(41)	24(24)	18(18)	25(25)	0(0)	22
	cchange	60(30)	62(25)	59(26)	36(26)	72(29)	56	40(40)	23(23)	20(20)	26(26)	0(0)	22
	Spike Slab	69(39)	73(34)	70(34)	46(40)	76(35)	66	31(31)	17(17)	16(16)	22(22)	0(0)	17
	Lasso	68(40)	74(34)	71(36)	47(40)	78(33)	67	32(32)	17(17)	16(16)	23(23)	0(0)	18
1	BLRM	33(15)	24(5)	23(5)	27(16)	60(25)	31	13(13)	27(27)	25(25)	25(25)	22(22)	22
	BLRMc1	82(9)	78(13)	77(14)	78(14)	80(12)	79	11(11)	13(13)	12(12)	10(10)	8(8)	11
	BLRMc2	79(8)	73(13)	70(15)	70(15)	74(13)	73	12(12)	15(15)	13(13)	12(12)	10(10)	12
	cchange	80(9)	73(13)	71(14)	72(13)	75(11)	74	11(11)	15(15)	14(14)	11(11)	10(10)	12
	Spike Slab	82(9)	78(13)	75(15)	74(16)	76(14)	77	12(12)	13(13)	11(11)	10(10)	11(11)	11
	Lasso	79(8)	73(14)	69(16)	70(15)	75(13)	73	12(12)	14(14)	12(12)	14(14)	13(13)	13
2	BLRM	31(15)	24(5)	22(5)	26(16)	58(24)	30	15(15)	27(27)	26(26)	26(26)	23(23)	23
	BLRMc2	79(8)	73(12)	71(15)	71(15)	74(13)	74	12(12)	15(15)	13(13)	12(12)	10(10)	12
	cchange	79(9)	73(12)	72(15)	71(14)	73(11)	73	12(12)	15(15)	13(13)	12(12)	12(12)	13
	Spike Slab	78(9)	71(14)	67(16)	65(16)	71(12)	70	13(13)	15(15)	13(13)	14(14)	14(14)	14
	Lasso	78(9)	70(14)	68(16)	68(16)	74(13)	71	12(12)	15(15)	13(13)	15(15)	14(14)	14

Table A.2: Average proportion of patients (out of 100) assigned to their pMTD at the end of the trial and to an overdose for inclusion of two continuous covariates under different scenarios to simulate the data, with zero, one or two continuous covariates. In parenthesis are given the standard deviations.

Covariates	Model	MTD.trial						Ov.trial					
		d1	d2	d3	d4	d5	Mean	d1	d2	d3	d4	d5	Mean
0	BLRM	61(33)	53(23)	56(23)	30(21)	47(17)	48	39(33)	29(25)	17(21)	18(20)	0(0)	21
	BLRMc	53(23)	44(15)	46(17)	20(12)	39(15)	38	47(23)	32(18)	18(16)	18(16)	0(0)	23
	cchange	53(23)	44(15)	43(17)	23(13)	44(15)	40	47(23)	32(19)	21(18)	21(17)	0(0)	24
	Spike Slab	58(27)	50(19)	47(19)	26(16)	45(16)	44	42(27)	28(20)	19(19)	19(18)	0(0)	22
	Lasso	58(28)	51(19)	49(21)	28(17)	46(16)	45	42(28)	28(22)	20(20)	19(18)	0(0)	22
1	BLRM	29(15)	23(8)	20(8)	22(11)	37(14)	26	17(15)	28(8)	23(7)	19(7)	14(6)	20
	BLRMc1	61(10)	53(10)	47(10)	45(9)	46(10)	50	12(9)	20(10)	14(8)	11(7)	8(6)	13
	BLRMc2	58(10)	48(10)	41(10)	38(10)	41(11)	45	13(8)	21(9)	14(7)	11(6)	8(5)	13
	cchange	58(10)	48(10)	43(10)	41(10)	44(10)	47	13(8)	21(9)	15(7)	11(6)	9(6)	14
	Spike Slab	60(10)	52(10)	44(10)	40(10)	43(11)	47	14(8)	20(9)	14(8)	12(7)	9(6)	14
	Lasso	57(11)	47(10)	39(10)	38(10)	43(10)	44	14(9)	21(9)	16(7)	14(7)	11(6)	15
2	BLRM	29(15)	23(8)	19(7)	22(11)	37(14)	25	18(15)	28(8)	23(7)	19(7)	14(6)	20
	BLRMc2	58(10)	48(10)	42(10)	39(10)	41(10)	45	14(8)	20(9)	15(7)	11(7)	8(5)	14
	cchange	57(10)	48(10)	42(10)	41(10)	43(9)	46	14(9)	21(9)	16(8)	12(7)	9(6)	14
	Spike Slab	56(11)	46(10)	38(10)	36(10)	42(10)	43	15(9)	21(8)	16(7)	14(7)	10(6)	15
	Lasso	55(11)	45(10)	38(10)	37(10)	42(10)	43	14(9)	21(8)	17(7)	15(7)	11(6)	16

Table A.3: Average proportion of patients (out of 100) assigned to their pMTD during the trial and to an overdose for inclusion of two continuous covariates under different scenario to simulate the data, with zero, one or two continuous covariates. In parenthesis are given the standard deviations.

During the trial, when no covariates were used to generate the data, Spike and Slab and LASSO have respectively 44% and 45% of pMTD correct allocation, the highest difference with BLRM being in scenario d_3 (56% for BLRM against 47% and 49% for Spike and Slab and LASSO). For the overdosing during the trial, all methods performs equally for scenario d_5, while the Spike and Slab and the LASSO perform better than the BLRM for the scenario d_2 (28% vs 29% for BLRM), and the BLRMc performs equally than the BLRM for scenario d_4.
When one normal covariate was used to generate the data, the BLRMc1 show better performance in term of correct pMTD allocation during the trial, outperforming all other models in average (50%) and scenario by scenario, followed by c_{change} and Spike and Slab (47% and BLRMc2 and LASSO (45% and 44%). The Mean of overdosing during the trial is the same than the BLRMc2 with two covariates (13%), being lower only for scenario d_1 (12% vs 13%) and being either equal or lower for other models scenario by scenario.
When two normal covariates were used to generate the data, the geometric Mean of percentage of correct pMTD allocation during the trial of BLRMc2 is lower than the c_{change} (45% vs 46%) and superior only for scenario d_1 (58% vs 57%), but all methods (except BLRM give similar pMTD allocation during the trial in average, as well as overdosing (except BLRM: 20% against 14% for BLRMc2 and c_{change}, the lowest). Spike and Slab and LASSO performed better in pMTD allocation during the tial for scenario d_5 (42% vs 41%).
The given standard deviations for the MTD.add metric show that in the setting where no covariates was used to generate the data, c_{change} and BLRMc tend to have the same standard deviations in term of pMTD allocations, these standard deviations are lower than the one obtained with Spike and Slab and LASSO (similar standard deviations), and the highest values for standard deviations are obtained with the BLRM. In the setting where one or two covariates were used to generate the data, BLRMc1, BLRMc2, c_{change}, Spike and Slab and LASSO show similar standard deviations between scenarios, and lower (or similar in scenario d_4) than BLRM.

1.2 Case of one normal and one binary covariate included

Covariates	Model	MTD.add						Ov.add					
		d1	d2	d3	d4	d5	Mean	d1	d2	d3	d4	d5	Mean
0	BLRM	70(46)	80(40)	78(41)	49(50)	83(38)	71	30(30)	14(14)	12(12)	20(20)	0(0)	15
	BLRMc	62(30)	63(26)	61(27)	37(28)	67(30)	57	38(38)	23(23)	19(19)	23(23)	0(0)	21
	cchange	62(30)	63(26)	61(28)	38(29)	68(30)	57	38(38)	24(24)	19(19)	24(24)	0(0)	21
	Spike Slab	68(38)	72(34)	71(33)	46(40)	71(37)	65	32(32)	18(18)	14(14)	20(20)	0(0)	17
	Lasso	68(37)	72(32)	70(34)	47(38)	74(34)	65	32(32)	19(19)	16(16)	22(22)	0(0)	18
1b	BLRM	48(12)	48(11)	12(22)	27(25)	77(42)	36	16(16)	8(8)	44(44)	36(36)	0(0)	21
	BLRMc1	64(28)	56(36)	45(33)	52(32)	70(37)	57	21(21)	12(12)	38(38)	23(23)	0(0)	19
	BLRMc2	56(28)	53(31)	34(30)	48(24)	70(37)	51	29(29)	19(19)	41(41)	24(24)	0(0)	23
	cchange	59(22)	52(28)	44(26)	46(24)	69(34)	53	24(24)	15(15)	36(36)	24(24)	0(0)	20
	Spike Slab	59(22)	49(32)	44(30)	42(27)	72(37)	52	19(19)	15(15)	35(35)	27(27)	0(0)	19
	Lasso	58(22)	54(31)	39(30)	42(25)	73(36)	52	18(18)	13(13)	38(38)	29(29)	0(0)	20
1c	BLRM	32(15)	24(5)	22(4)	27(16)	59(25)	31	13(13)	27(27)	25(25)	25(25)	21(21)	22
	BLRMc1	82(9)	78(14)	78(14)	78(14)	80(13)	79	11(11)	13(13)	12(12)	11(11)	8(8)	11
	BLRMc2	80(8)	74(13)	73(15)	74(14)	77(13)	75	12(12)	15(15)	13(13)	12(12)	9(9)	12
	cchange	80(8)	74(13)	74(14)	74(14)	76(14)	76	12(12)	15(15)	12(12)	11(11)	9(9)	12
	Spike Slab	81(9)	78(13)	76(15)	74(15)	76(14)	77	12(12)	12(12)	10(10)	11(11)	10(10)	11
	Lasso	81(9)	76(14)	74(15)	74(16)	78(13)	76	12(12)	13(13)	12(12)	12(12)	11(11)	12
2mix	BLRM	41(18)	42(12)	26(5)	27(12)	57(25)	37	17(17)	24(24)	26(26)	25(25)	22(22)	23
	BLRMc2	64(13)	58(16)	62(17)	63(17)	71(16)	64	20(20)	21(21)	15(15)	14(14)	11(11)	16
	cchange	64(13)	58(16)	62(17)	62(16)	70(15)	63	20(20)	21(21)	15(15)	15(15)	12(12)	17
	Spike Slab	59(14)	52(15)	55(16)	58(17)	70(16)	58	20(20)	22(22)	17(17)	17(17)	14(14)	18
	Lasso	59(14)	53(15)	53(16)	58(16)	71(16)	59	19(19)	22(22)	19(19)	18(18)	15(15)	19

Table A.4: Average proportion of patients (out of 100) assigned to their pMTD at the end of the trial and to an overdose for inclusion of two covariates (one continuous and one binary) in term of MTD and overdosing under different scenarios to simulate the data, with zero covariate to simulate the data, one binary covariate (1b), one continuous continuous (1c) or two covariates (one continuous and one binary) to generate the data (2mix).

Covariates	Model	MTD.trial						Ov.trial					
		d1	d2	d3	d4	d5	Mean	d1	d2	d3	d4	d5	Mean
0	BLRM	61(33)	53(23)	56(23)	30(21)	47(17)	48	39(33)	29(25)	17(21)	18(20)	0(0)	21
	BLRMc	56(24)	45(16)	46(17)	23(14)	39(16)	40	44(24)	32(19)	18(17)	18(16)	0(0)	22
	cchange	56(24)	45(16)	44(17)	24(14)	42(16)	41	44(24)	32(19)	20(18)	19(17)	0(0)	23
	Spike Slab	59(27)	49(19)	48(19)	28(17)	44(17)	44	41(27)	29(21)	18(19)	18(17)	0(0)	21
	Lasso	60(27)	50(19)	49(19)	28(16)	45(16)	45	40(27)	30(22)	20(19)	19(18)	0(0)	22
1b	BLRM	45(13)	40(9)	17(12)	25(13)	44(20)	32	23(24)	16(14)	37(11)	24(14)	0(0)	20
	BLRMc1	56(18)	46(17)	32(14)	33(14)	42(19)	41	25(22)	16(14)	32(16)	19(14)	0(0)	18
	BLRMc2	47(17)	45(15)	25(14)	33(10)	42(18)	37	37(22)	21(13)	35(14)	19(13)	0(0)	22
	cchange	51(17)	43(15)	30(12)	31(12)	41(18)	38	29(21)	17(13)	32(15)	19(12)	0(0)	19
	Spike Slab	52(15)	43(16)	30(14)	29(12)	42(19)	38	26(21)	17(14)	31(15)	20(13)	0(0)	19
	Lasso	51(15)	44(15)	28(13)	29(11)	42(19)	38	25(22)	16(13)	33(14)	21(13)	0(0)	19
1c	BLRM	29(15)	23(8)	20(7)	22(12)	37(14)	26	18(15)	28(8)	23(7)	19(7)	14(6)	20
	BLRMc1	61(10)	53(10)	47(10)	45(10)	47(10)	50	13(9)	19(10)	15(8)	11(7)	8(6)	13
	BLRMc2	59(10)	51(10)	44(10)	41(10)	43(11)	47	13(9)	20(9)	15(8)	11(7)	8(5)	13
	cchange	59(10)	51(10)	45(10)	43(10)	44(10)	48	13(9)	20(9)	15(8)	12(7)	8(5)	14
	Spike Slab	60(10)	52(11)	45(10)	41(10)	44(10)	48	14(9)	19(10)	14(8)	12(7)	9(6)	14
	Lasso	58(11)	49(10)	42(10)	40(11)	44(10)	47	14(9)	20(9)	16(8)	13(7)	10(6)	15
2mix	BLRM	39(16)	34(11)	23(7)	23(10)	35(14)	30	22(19)	28(15)	23(9)	19(8)	14(7)	21
	BLRMc2	53(13)	44(12)	38(10)	36(11)	39(12)	42	22(15)	25(14)	16(10)	13(8)	8(6)	17
	cchange	53(13)	44(12)	38(10)	37(10)	41(11)	42	22(15)	26(15)	17(10)	13(8)	9(6)	18
	Spike Slab	50(13)	41(11)	35(10)	35(10)	40(12)	40	22(15)	26(14)	18(10)	15(8)	11(6)	18
	Lasso	49(14)	41(11)	34(9)	35(10)	41(11)	39	22(16)	27(15)	20(10)	16(8)	11(6)	19

Table A.5: Average proportion of patients assigned to their pMTD during the trial and to an overdose for inclusion of two covariates (one continuous and one binary) under different scenarios to simulate the data, with zero covariate to generate the data, one binary covariate used to generate the data (1b), one continuous covariate (1c) or two covariates (one continuous and one binary) to generate the data (2mix).

In the setting where no covariates was used to generate the data, the pMTD allocations during the trial is the highest as well, being 8% higher than BLRMc on average (40% vs 48%) with a maximum difference of 10% for scenario d_3 (56% vs 46% for BLRMc). All methods perform similarly in term of overdosing during the trial, with an average of 21% for BLRM and never more than 2% of difference.
In the setting where one binary covariate was use to generate the data, BLRMc1 results in the highest pMTD allocations rate during the trial across all methods (41%), followed by c_{change}, Spike and Slab and LASSO (38%) with a maximum difference with LASSO for scenario d_1 (51% vs 56%). The overdosing using BLRMc1 during the trials is the lowest and comparable to LASSO, Spike and Slab and c_{change} (19%). The BLRM show the worst results in term of pMTD allocations during the trial (32% vs 41% for BLRMc1) but comparable overdosing to other methods.
In the setting where one normal and one binary covariate were used to generate the data, the pMTD allocations rate is similar between all methods during the trial, with never more than 4% of difference (except BLRM with at least 9% less on average than other methods). The given standard deviations for the MTD.add metric show that in the setting where no covariates was used to generate the data, c_{change} and BLRMc have comparable standard deviations in term of pMTD allocations, these standard deviations are lower than the one obtained with Spike and Slab and LASSO (similar standard deviations), and the highest values for standard deviations are obtained with the BLRM. In the setting where one binary covariate was used to generate the data, BLRMc1, BLRMc2, c_{change}, Spike and Slab and LASSO show similar standard deviations between scenarios (BLRMc1 and BLRMc2 have higher standard deviation for scenario d_1 and d_4) and higher than BLRM (except for scenario d_4 and d_5: comparable and higher for BLRM). In the setting where one continuous covariate or one continuous and one binary covariates was used to generate the data, BLRMc1, BLRMc2, Spike and Slab and LASSO show similar standard deviation, lower higher than the BLRM ones except for scenario d_1 and d_5, for each setting.

Correlattion	Model	MTD.add						Ov.add					
		d1	d2	d3	d4	d5	Mean	d1	d2	d3	d4	d5	Mean
-0.8	BLRMc2	72(11)	57(15)	58(17)	61(19)	69(18)	63	12(12)	19(19)	17(17)	15(15)	12(12)	15
	Spike Slab	73(12)	53(16)	58(19)	56(19)	67(19)	61	14(14)	20(20)	15(15)	16(16)	15(15)	16
	LASSO	70(14)	50(16)	54(19)	54(19)	69(19)	59	13(13)	21(21)	17(17)	18(18)	16(16)	17
	BLRMc1	33(11)	32(6)	26(5)	31(11)	60(20)	35	18(18)	25(25)	24(24)	23(23)	18(18)	22
-0.5	BLRMc2	74(10)	61(15)	68(15)	64(17)	72(17)	68	14(14)	19(19)	15(15)	14(14)	10(10)	14
	Spike Slab	70(13)	54(17)	68(16)	57(18)	69(17)	63	15(15)	21(21)	13(13)	16(16)	14(14)	16
	LASSO	70(13)	54(16)	66(17)	57(19)	71(17)	63	14(14)	20(20)	13(13)	17(17)	15(15)	16
	BLRMc1	42(10)	33(6)	27(7)	36(12)	63(18)	39	17(17)	25(25)	25(25)	23(23)	17(17)	21
-0.2	BLRMc2	77(9)	58(16)	70(15)	74(13)	72(17)	70	13(13)	22(22)	13(13)	13(13)	10(10)	14
	Spike Slab	75(10)	50(14)	66(16)	70(15)	70(16)	65	14(14)	24(24)	12(12)	12(12)	14(14)	15
	LASSO	75(11)	52(16)	66(16)	71(15)	72(17)	67	13(13)	22(22)	13(13)	13(13)	14(14)	15
	BLRMc1	46(9)	43(9)	33(8)	36(13)	66(16)	43	16(16)	25(25)	24(24)	24(24)	16(16)	21
0.2	BLRMc2	79(8)	59(15)	71(15)	67(17)	73(15)	70	12(12)	22(22)	13(13)	12(12)	10(10)	14
	Spike Slab	77(9)	52(14)	66(16)	61(16)	72(15)	65	14(14)	23(23)	13(13)	15(15)	13(13)	16
	LASSO	78(9)	54(16)	68(16)	65(16)	74(14)	67	13(13)	21(21)	13(13)	15(15)	14(14)	15
	BLRMc1	55(8)	44(8)	41(9)	51(12)	69(15)	51	15(15)	24(24)	22(22)	20(20)	14(14)	19
0.5	BLRMc2	79(8)	63(15)	73(14)	71(14)	73(15)	72	12(12)	20(20)	13(13)	12(12)	10(10)	13
	Spike Slab	77(9)	56(13)	67(14)	67(13)	72(14)	67	13(13)	22(22)	14(14)	14(14)	13(13)	15
	LASSO	78(9)	57(16)	70(15)	69(15)	74(14)	69	12(12)	20(20)	13(13)	14(14)	14(14)	15
	BLRMc1	64(8)	50(9)	50(9)	60(11)	71(13)	58	14(14)	23(23)	20(20)	17(17)	13(13)	17
0.8	BLRMc2	81(8)	77(11)	75(13)	73(15)	72(17)	75	12(12)	14(14)	12(12)	11(11)	9(9)	12
	Spike Slab	78(7)	73(11)	71(12)	71(13)	72(17)	73	13(13)	15(15)	13(13)	13(13)	12(12)	13
	LASSO	80(8)	74(12)	73(14)	71(15)	74(16)	74	12(12)	14(14)	13(13)	13(13)	13(13)	13
	BLRMc1	72(6)	62(8)	62(10)	65(11)	73(15)	67	13(13)	18(18)	17(17)	15(15)	11(11)	15
0.95	BLRMc2	80(8)	79(12)	77(14)	74(15)	73(19)	77	13(13)	13(13)	11(11)	11(11)	9(9)	11
	Spike Slab	80(8)	78(11)	76(13)	74(15)	73(19)	76	13(13)	13(13)	11(11)	11(11)	11(11)	12
	LASSO	80(9)	78(12)	75(15)	73(16)	75(17)	76	12(12)	13(13)	12(12)	12(12)	12(12)	12
	BLRMc1	77(7)	73(10)	73(11)	73(13)	75(18)	74	13(13)	15(15)	14(14)	13(13)	9(9)	13

Table A.6: Average proportion of patients (out of 100) assigned to their pMTD at the end of the trial and to an overdose for inclusion of two correlated normal covariates under different scenarios to simulate the data, with zero, one or two continuous covariates. Several correlations, negative and positive, were considered. In parenthesis are given the standard deviations.

Correlattion	Model	MTD.trial						Ov.trial					
		d1	d2	d3	d4	d5	Mean	d1	d2	d3	d4	d5	Mean
-0.8	BLRMc2	49(11)	40(10)	33(9)	32(10)	37(12)	38	16(10)	25(11)	18(8)	14(7)	9(6)	16
	Spike Slab	48(12)	37(10)	32(10)	31(10)	39(13)	37	17(10)	26(11)	18(8)	16(8)	10(6)	18
	LASSO	46(12)	36(9)	30(9)	30(10)	39(12)	36	18(10)	26(11)	20(8)	17(8)	11(6)	18
	BLRMc1	30(12)	29(9)	23(8)	24(10)	37(13)	28	21(12)	28(11)	22(8)	18(8)	11(6)	20
-0.5	BLRMc2	54(12)	43(11)	39(10)	35(10)	39(12)	41	17(11)	24(12)	16(7)	13(7)	8(6)	16
	Spike Slab	51(12)	39(10)	37(10)	32(10)	40(12)	39	18(11)	26(11)	17(7)	16(8)	10(6)	17
	LASSO	50(13)	38(10)	35(10)	32(10)	41(12)	39	17(11)	25(11)	18(7)	16(8)	11(6)	18
	BLRMc1	37(13)	30(9)	23(8)	26(10)	39(12)	30	20(12)	27(11)	22(7)	18(8)	11(6)	20
-0.2	BLRMc2	56(11)	42(11)	40(10)	42(10)	39(12)	43	15(9)	27(13)	15(7)	14(7)	7(6)	16
	Spike Slab	54(11)	39(10)	37(9)	39(10)	41(12)	42	17(9)	28(13)	16(8)	14(7)	10(6)	17
	LASSO	53(11)	40(10)	37(10)	39(11)	42(11)	42	16(10)	27(14)	17(8)	15(7)	11(6)	17
	BLRMc1	39(12)	36(10)	26(9)	26(11)	40(12)	33	19(10)	28(13)	21(7)	20(7)	10(6)	20
0.2	BLRMc2	58(10)	43(11)	43(10)	38(11)	40(12)	44	14(8)	27(13)	14(8)	11(7)	7(5)	15
	Spike Slab	56(10)	40(10)	40(10)	36(10)	42(12)	42	16(8)	27(13)	15(8)	14(8)	9(6)	16
	LASSO	56(11)	39(10)	39(10)	36(10)	43(11)	42	15(9)	27(13)	17(7)	15(8)	10(6)	17
	BLRMc1	44(12)	36(9)	30(10)	33(11)	41(12)	36	17(9)	28(12)	20(7)	16(7)	10(6)	18
0.5	BLRMc2	60(10)	45(11)	44(10)	40(11)	40(12)	45	14(9)	25(13)	14(8)	11(7)	7(6)	14
	Spike Slab	58(10)	42(10)	42(10)	39(10)	42(11)	44	15(9)	26(12)	15(8)	12(7)	9(6)	16
	LASSO	57(11)	42(11)	41(10)	38(10)	43(11)	44	14(9)	26(13)	16(8)	13(7)	11(6)	16
	BLRMc1	50(11)	39(9)	34(10)	37(11)	42(11)	40	15(9)	26(12)	18(7)	14(7)	10(6)	17
0.8	BLRMc2	61(9)	53(10)	46(10)	41(10)	39(12)	47	13(8)	20(9)	14(8)	11(7)	7(6)	13
	Spike Slab	60(10)	52(10)	45(10)	41(10)	42(12)	47	14(8)	20(9)	14(8)	13(8)	9(6)	14
	LASSO	59(10)	50(10)	43(10)	39(10)	42(12)	46	13(8)	20(9)	16(8)	14(8)	10(6)	15
	BLRMc1	55(10)	45(9)	40(10)	40(10)	43(12)	44	14(8)	22(9)	17(7)	14(7)	8(6)	15
0.95	BLRMc2	61(10)	54(10)	47(10)	42(11)	39(13)	48	15(9)	19(9)	13(8)	11(7)	6(6)	13
	Spike Slab	60(10)	55(10)	48(10)	43(10)	42(13)	49	15(9)	18(9)	13(8)	12(8)	8(6)	13
	LASSO	59(10)	52(10)	45(10)	40(10)	43(12)	47	15(9)	19(9)	15(8)	14(8)	9(6)	14
	BLRMc1	58(10)	51(10)	46(9)	43(9)	43(12)	48	14(9)	20(9)	16(8)	13(8)	7(6)	14

Table A.7: Average proportion of patients (out of 100) assigned to their pMTD during the trial and to an overdose for inclusion of two correlated normal covariates under different scenarios to simulate the data, with zero, one or two continuous covariates. Several correlations, negative and positive, were considered. In parenthesis are given the standard deviations.

2 Calibration analysis for c_{change} criterion for several combinations, case of two covariates inclusion (one binary and one continuous)

Covariates	0					1 binary					1 continuous					2 (one binary and one continuous)					
Combos	d1	d2	d3	d4	d5	d1	d2	d3	d4	d5	d1	d2	d3	d4	d5	d1	d2	d3	d4	d5	Mean
0.3-0.3	62.60	62.07	61.10	36.49	66.64	58.90	52.28	43.75	46.32	69.53	79.81	74.03	73.39	72.28	68.90	63.84	57.60	61.59	62.32	69.12	61.11
0.2-0.3	62.25	62.84	60.96	37.29	67.42	58.87	51.99	43.60	46.16	69.36	79.96	74.38	73.96	73.27	73.33	64.14	57.85	61.56	62.49	70.23	61.54
0.1-0.3	61.97	62.68	61.16	37.90	67.52	58.96	51.88	43.54	46.13	69.36	79.87	74.42	74.29	73.87	76.24	64.04	58.03	61.60	62.30	70.50	**61.74**
0.3-0.2	62.52	61.68	60.83	35.87	64.58	58.67	52.42	43.39	46.36	69.35	79.80	73.94	72.98	72.02	67.41	63.95	57.42	61.79	62.70	68.91	60.81
0.2-0.2	62.31	62.62	60.70	36.73	65.51	58.62	52.18	43.50	46.24	69.16	79.91	74.33	73.59	72.89	71.90	64.17	57.59	61.76	63.15	70.26	61.31
0.1-0.2	62.08	62.49	61.15	37.44	66.18	58.60	52.08	43.49	46.21	69.16	79.79	74.37	73.84	73.50	75.31	64.10	57.78	61.87	63.03	70.76	61.59
0.3-0.1	62.49	61.56	60.90	35.75	63.56	58.54	52.23	43.46	46.32	69.45	79.77	73.81	72.70	71.46	66.70	64.10	57.51	61.43	62.33	68.58	60.62
0.2-0.1	62.19	62.38	60.83	36.62	64.74	58.47	51.98	43.58	46.28	69.27	79.87	74.23	73.44	72.76	70.45	64.30	57.70	61.51	62.98	69.59	61.13
0.1-0.1	61.91	62.48	61.02	37.31	65.69	58.75	52.16	43.55	46.30	69.25	79.77	74.30	73.71	73.58	74.47	64.10	57.84	61.91	62.96	70.54	61.52

Table A.9: Calibration analysis for c_{change} criterion for several combinations of the criterion for the continuous and binary covariate. The results are the percentage of correct MTD allocation for additional patients at the end of the trial in different scenario to simulate the data, with zero, one or two covariates.

3 Calibration analysis for several combinations of LASSO parameter δ, case of two covariates inclusion (one binary and one continuous)

Covariates	0					1 binary					1 continuous					2 (one binary and one continuous)					
Combos	d1	d2	d3	d4	d5	d1	d2	d3	d4	d5	d1	d2	d3	d4	d5	d1	d2	d3	d4	d5	Mean
0.2-0.2	69.52	70.87	71.25	49.47	77.27	54.22	51.19	32.27	34.24	75.72	79.77	73.87	69.88	69.86	74.30	53.72	46.32	45.99	49.62	66.36	58.88
0.4-0.2	66.61	67.82	67.46	46.64	75.53	54.05	50.97	36.68	38.44	77.05	80.03	74.67	71.17	70.19	75.05	55.32	47.78	47.57	53.27	68.54	59.70
1-0.2	65.16	68.61	64.58	47.47	74.97	55.51	51.43	39.65	42.85	74.60	80.01	74.46	72.01	71.52	75.75	56.99	50.17	49.61	55.60	69.59	60.78
0.2-0.4	67.20	69.91	68.07	47.38	76.94	51.37	50.23	35.13	34.69	73.29	81.03	74.88	71.34	68.14	73.52	55.01	47.02	47.60	52.37	67.06	58.89
0.4-0.4	69.29	75.38	70.89	47.43	73.95	55.72	52.35	34.91	36.55	74.86	79.96	74.03	71.63	71.42	75.59	56.92	50.68	51.26	55.69	69.67	60.79
1-0.4	67.66	72.23	69.76	46.71	74.24	58.44	53.62	39.11	42.10	72.96	80.82	75.91	74.25	73.56	77.97	58.97	53.25	52.95	58.27	70.91	**62.39**
0.2-1	67.67	67.44	66.29	45.81	74.42	51.52	50.27	37.04	34.80	72.91	81.33	76.20	72.54	71.10	74.06	56.37	49.03	50.15	54.16	68.10	59.45
0.4-1	68.79	72.43	69.49	46.91	74.24	56.22	52.20	38.05	38.73	70.63	81.16	76.78	73.16	72.43	76.35	58.06	52.74	53.53	58.00	70.46	61.62
1-1	68.95	72.96	70.74	46.09	72.42	56.45	52.35	35.98	38.68	71.66	80.35	74.43	72.01	72.92	77.54	60.14	54.37	55.56	59.61	71.40	61.79

Table A.10: Calibration analysis for several combinations of the LASSO hyperparameter δ for the continuous and binary covariate. The results are the percentage of correct MTD allocation for additional patients at the end of the trial in different scenario to simulate the data, with zero, one or two covariates.

4 Calibration analysis for several combinations of Spike and Slab variance parameter ν, case of two covariates inclusion (one binary and one continuous)

Covariates	0					1 binary					1 continuous					2 (one binary and one continuous)					
Combos	d1	d2	d3	d4	d5	d1	d2	d3	d4	d5	d1	d2	d3	d4	d5	d1	d2	d3	d4	d5	Mean
0.025-0.025	68.47	73.73	68.80	46.82	71.19	58.21	50.95	42.68	42.24	71.15	81.45	78.03	75.60	73.84	75.75	59.14	52.38	54.38	57.28	69.06	62.35
0.05-0.025	67.18	72.28	69.78	45.34	71.78	59.20	50.10	43.26	41.37	72.03	81.25	78.04	75.50	73.60	76.30	58.32	51.91	54.28	57.30	69.49	62.16
0.1-0.025	67.94	72.72	68.90	45.08	71.22	58.82	50.84	41.76	41.06	71.35	81.53	77.82	75.18	73.05	75.67	58.74	52.38	54.67	57.38	69.43	62.01
0.025-0.05	69.18	71.66	69.33	45.21	71.39	58.98	51.04	42.95	42.04	72.33	81.31	78.06	75.59	73.60	76.13	58.90	52.04	54.56	57.48	69.03	62.31
0.05-0.05	68.39	72.48	70.16	45.67	71.17	58.61	50.97	41.58	41.73	71.68	81.43	78.04	75.29	73.37	76.40	58.89	52.02	54.97	57.51	69.39	62.22
0.1-0.05	67.94	71.79	70.93	46.45	71.50	59.15	49.25	43.64	42.07	71.55	81.47	78.15	75.70	73.87	76.04	59.01	52.27	54.58	57.54	69.92	**62.42**
0.025-0.1	68.05	72.67	69.60	46.37	71.69	59.30	50.36	42.33	42.04	71.53	81.39	78.07	75.56	73.22	75.95	58.78	51.73	54.52	57.16	69.46	62.25
0.05-0.1	68.33	72.08	70.73	44.91	71.43	58.61	49.96	42.96	41.10	71.06	81.49	77.98	75.33	73.76	76.11	58.93	51.93	54.61	57.40	69.47	62.13
0.1-0.1	67.60	72.10	70.27	45.73	71.61	58.68	49.43	42.70	42.43	70.58	81.45	78.01	75.39	73.66	76.13	58.77	52.01	54.98	57.40	69.56	62.19

Table A.11: Calibration analysis for several combinations of of Spike and Slab variance parameter ν, case of two covariates inclusion (one binary and one continuous). The results are the percentage of correct MTD allocation for additional patients at the end of the trial in different scenario to simulate the data, with zero, one or two covariates.

5 Covariates inclusion with the c_{change} criterion

The proportion of trials including at least one of the two normal covariates with the c_{change} criterion is given in Table A.12. The proportion of inclusion of at least one covariate, even in the case where no covariate was significant, if higher than 77%. This show that the c_{change} criterion tends to include no-significant covariates.

Significant covariates	% of trials with at least one covariate included				
	d1	d2	d3	d4	d5
0	0.99	0.99	0.98	0.97	0.77
1	1.00	1.00	0.99	0.99	0.87
2	1.00	1.00	0.99	0.98	0.88

Table A.12: Proportion trials with at least one covariate included during the trial using the c_{change} criterion, over 1000 trials, when zero covariate, one normal or two normal covariates where significant, for each scenario.

The proportion of trials including at least one of the two covariates (one normal and one binary) with the c_{change} criterion is given in Table A.13. The proportion of inclusion of at least one covariate, even in the case where no covariate was significant, if higher than 90%. This show that the c_{change} criterion tends to include no-significant covariates.

Significant covariates	% of trials with at least one covariate inluded				
	d1	d2	d3	d4	d5
0	0.99	1.00	0.98	0.98	0.90
1b	0.97	0.99	0.98	0.96	0.76
1c	1.00	1.00	1.00	0.99	0.96
2m	1.00	1.00	0.99	0.99	0.93

Table A.13: Proportion trials with at least one covariate included during the trial using the c_{change} criterion, over 1000 trials, when zero covariate, one normal covariate (1c), one binary covariate (1b) or two covariates (2mix: one normal and one binary) where significant, for each scenario.

6 Sensitivity analysis (Emax model)

Scenario	MTD	$p_T(d_1)$	$p_T(d_2)$	$p_T(d_3)$	$p_T(d_4)$	$p_T(d_5)$
1	d_1	**0.24**	0.48	0.57	0.6	0.62
2	d_2	0.06	**0.25**	0.41	0.51	0.58
3	d_3	0.04	0.15	**0.26**	0.33	0.38
4	d_4	0.02	0.1	0.18	**0.25**	0.31
5	d_5	0.02	0.07	0.13	0.2	**0.25**

Table 13: Probabilities of toxicity per dose when no covariates were used to generate the data in the Emax model.

Covariates	Model	MTD.add						Ov.add					
		d1	d2	d3	d4	d5	Mean	d1	d2	d3	d4	d5	Mean
0	BLRM	86(34)	67(47)	49(50)	26(44)	58(49)	53	14(14)	26(26)	31(31)	32(32)	0(0)	20
	BLRMc	72(26)	50(25)	36(25)	20(18)	47(33)	41	28(28)	33(33)	32(32)	29(29)	0(0)	24
	c_{change}	81(33)	59(41)	41(41)	23(35)	58(44)	48	19(19)	30(30)	35(35)	35(35)	0(0)	24
	Spike Slab	83(30)	61(38)	39(37)	24(31)	52(42)	47	17(17)	27(27)	34(34)	33(33)	0(0)	22
	Lasso	83(31)	62(38)	40(38)	26(33)	56(43)	50	17(17)	28(28)	34(34)	33(33)	0(0)	22
1	BLRM	66(18)	35(12)	18(10)	30(18)	41(25)	35	8(8)	29(29)	32(32)	30(30)	27(27)	25
	BLRMc1	71(16)	56(20)	63(18)	55(22)	61(22)	61	20(20)	26(26)	19(19)	17(17)	13(13)	19
	BLRMc2	67(15)	50(16)	57(16)	49(19)	54(21)	55	24(24)	28(28)	19(19)	17(17)	14(14)	20
	c_{change}	65(16)	42(18)	46(25)	41(23)	50(25)	48	15(15)	31(31)	24(24)	24(24)	21(21)	23
	Spike Slab	68(15)	48(19)	57(20)	48(22)	55(22)	55	16(16)	28(28)	20(20)	21(21)	17(17)	20
	Lasso	69(14)	45(16)	52(20)	47(20)	55(21)	53	15(15)	28(28)	23(23)	23(23)	20(20)	22
2	BLRM	64(16)	28(8)	19(10)	28(20)	42(28)	33	7(7)	29(29)	33(33)	30(30)	27(27)	25
	BLRMc2	68(13)	54(16)	54(17)	57(17)	62(18)	59	22(22)	25(25)	21(21)	16(16)	13(13)	20
	c_{change}	64(13)	40(16)	40(21)	45(21)	55(22)	48	13(13)	30(30)	28(28)	24(24)	21(21)	23
	Spike Slab	66(12)	46(15)	47(17)	51(17)	58(18)	53	16(16)	26(26)	25(25)	21(21)	18(18)	21
	Lasso	68(12)	48(16)	47(18)	52(19)	60(19)	55	14(14)	25(25)	25(25)	22(22)	19(19)	21

Table A.14: Average proportion of patients (out of 100) assigned to their pMTD at the end of the trial and to an overdose for inclusion of two continuous covariates under different scenarios to simulate the data in the Emax model, with zero, one or two continuous covariates. In parenthesis are given the standard deviations.

Covariates	Model	MTD.trial						Ov.trial					
		d1	d2	d3	d4	d5	Mean	d1	d2	d3	d4	d5	Mean
0	BLRM	71(27)	46(25)	38(27)	21(19)	30(24)	38	29(27)	35(29)	25(27)	19(22)	0(0)	22
	BLRMc	61(21)	38(15)	31(18)	14(10)	25(19)	31	39(21)	36(20)	24(19)	18(17)	0(0)	23
	c_{change}	68(26)	41(23)	34(24)	18(16)	32(23)	35	32(26)	37(25)	28(25)	22(21)	0(0)	24
	Spike Slab	67(23)	43(20)	31(20)	17(14)	28(22)	34	33(23)	33(23)	26(22)	20(19)	0(0)	22
	Lasso	68(24)	44(21)	33(22)	19(16)	30(22)	35	32(24)	34(24)	26(24)	20(20)	0(0)	22
1	BLRM	57(18)	29(10)	17(8)	23(11)	26(14)	28	20(21)	32(15)	27(10)	22(10)	18(8)	24
	BLRMc1	60(15)	41(13)	40(13)	33(13)	36(14)	41	27(18)	30(17)	18(12)	14(12)	11(9)	20
	BLRMc2	57(14)	37(12)	36(12)	30(12)	31(13)	37	30(16)	30(15)	18(11)	13(10)	10(8)	20
	c_{change}	57(17)	33(11)	30(13)	28(13)	31(14)	35	23(21)	33(15)	23(11)	19(11)	15(9)	23
	Spike Slab	58(15)	36(12)	36(13)	30(12)	32(13)	37	26(18)	31(16)	19(11)	15(11)	12(9)	20
	Lasso	59(15)	35(11)	32(12)	29(12)	32(13)	36	24(18)	30(16)	21(11)	18(11)	14(9)	21
2	BLRM	56(17)	25(9)	18(8)	21(12)	26(15)	27	18(19)	30(13)	27(10)	22(9)	19(8)	23
	BLRMc2	57(14)	39(11)	34(12)	34(12)	35(12)	39	28(16)	27(13)	19(11)	13(9)	11(7)	20
	c_{change}	56(16)	31(10)	28(12)	29(12)	33(14)	34	21(20)	31(13)	24(11)	19(9)	16(8)	22
	Spike Slab	57(14)	35(10)	31(11)	31(12)	34(13)	37	24(16)	28(13)	21(11)	16(9)	13(8)	20
	Lasso	58(14)	35(10)	30(12)	31(12)	34(13)	37	23(17)	28(13)	22(11)	18(10)	14(8)	21

Table A.15: Average proportion of patients (out of 100) assigned to their pMTD during the trial and to an overdose for inclusion of two continuous covariates under different scenarios to simulate the data in the Emax model, with zero, one or two continuous covariates. In parenthesis are given the standard deviations.
